# Efficient Catalytic Oxidation of 3-Arylthio- and 3-Cyclohexylthio-lapachone Derivatives to New Sulfonyl Derivatives and Evaluation of Their Antibacterial Activities

**DOI:** 10.3390/molecules22020302

**Published:** 2017-02-16

**Authors:** Mariana F. do C. Cardoso, Ana T. P. C. Gomes, Caroline dos S. Moreira, Mário M. Q. Simões, Maria G. P. M. S. Neves, David R. da Rocha, Fernando de C. da Silva, Catarina Moreirinha, Adelaide Almeida, Vitor F. Ferreira, José A. S. Cavaleiro

**Affiliations:** 1 Department of Chemistry and QOPNA, University of Aveiro, Aveiro 3810-193, Portugal; marianafcc83@hotmail.com (M.F.d.C.C.); ana.peixoto@ua.pt (A.T.P.C.G.); msimoes@ua.pt (M.M.Q.S.); gneves@ua.pt (M.G.P.M.S.N.); 2 Departamento de Química Orgânica, Instituto de Química, Universidade Federal Fluminense, Niterói 24020-150, RJ, Brazil; carolmoreira13@gmail.com (C.d.S.M.); davidrrocha@vm.uff.br (D.R.d.R.); gqofernando@vm.uff.br (F.d.C.d.S.); 3 Department of Biology and CESAM, University of Aveiro, Aveiro 3810-193, Portugal; anacatarinafernandes@gmail.com (C.M.); aalmeida@ua.pt (A.A.)

**Keywords:** oxidation, porphyrinatoMn(III), hydrogen peroxide, arylthio/cyclohexylthio-lapachones, sulfonyl-lapachones, polyvinylpyrrolidone, antibacterial activity

## Abstract

New sulfonyl-lapachones were efficiently obtained through the catalytic oxidation of arylthio- and cyclohexylthio-lapachone derivatives with hydrogen peroxide in the presence of a Mn(III) porphyrin complex. The antibacterial activities of the non-oxidized and oxidized lapachone derivatives against the Gram-negative bacteria *Escherichia coli* and the Gram-positive bacteria *Staphylococcus aureus* were evaluated after their incorporation into polyvinylpyrrolidone (PVP) micelles. The obtained results show that the PVP-formulations of the lapachones **4b**–**g** and of the sulfonyl-lapachones **7e** and **7g** reduced the growth of *S. aureus*.

## 1. Introduction

**Figure 1:**
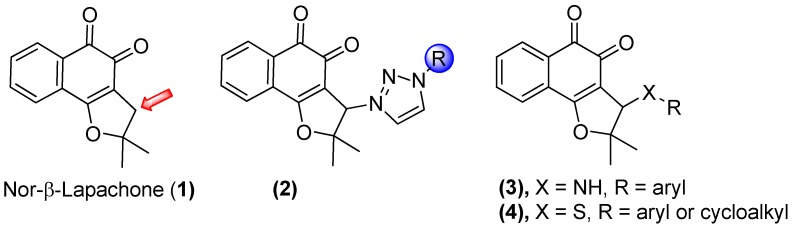
Bioactive nor-β-lapachone (**1**) and general structures of some derivatives.

Naphthoquinones have been proven to be good antibacterial [1,2,3], antifungal [4], antiprotozoal [5,6], and antiviral agents [7,8,9,10]. Nor-β-Lapachone (**1**) is a very important naphthoquinone since it has selective cytotoxicity to human lymphocytes, HL-60 leukemia cells and murine fibroblasts V79 [11]. This compound has been the basis for the synthesis of several important analogues or derivatives with improved biological activities [12,13,14,15,16,17]. Recently, it was demonstrated that the modification of the dihydrofuran ring of nor-β-lapachone (**1**) could considerably change its activity against cancer cells [18,19,20,21], *Trypanosoma cruzi* (*T. cruzi*) [22,23,24,25,26], and candidal agents [27]. Indeed, the triazolyl series of compounds **2** and the arylamine group of compounds **3** are very active against some cancer cell lines and *T. cruzi*, respectively (Figure 1).

Since derivatives containing sulfur groups are interesting compounds due to their significant properties as intermediates in many biological processes [28,29], our group recently reported a straightforward and efficient one-step reaction to prepare new nor-β-lapachone derivatives (**4**) tethered with thio-substituents at position 3 of the furan ring (Figure 1) [30].

**Figure 2:**
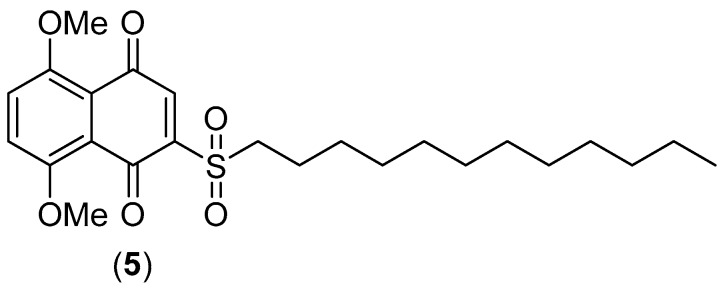
Naphthoquinone containing a sulfone moiety which is of biological significance.

It is known that sulfones are widely used as solvents, polymers, and biopharmaceutical agents. Indeed, several drug molecules containing sulfone groups are used for the treatment of leprosy, dermatitis herpetiformis, and tuberculosis. Moreover, sulfones have also demonstrated various biological activities, mainly as anti-inflammatory, antimicrobial, anticancer, anti-HIV, antimalarial, and anti-inflammatory [31] properties. In particular, sulfones conjugated to naphthoquinones have several biological activities reported in the literature. Lee and coworkers [32] reported a new series of naphthoquinone derivatives in which compound **5**, (Figure 2), proved to be a most potent inhibitor against HepG2 cell lines (IC_50_ of 0.44 µM).

Considering that the oxidation of organosulfur compounds can provide new derivatives with potential biological activities, and following our previous studies on oxidative transformations, we decided to evaluate the possibility of using a metalloporphyrin as a catalyst and hydrogen peroxide as oxidant in the oxidation process of 3-arylthio-nor-β-lapachone derivatives **4a**–**f**. This aryl-substituted lapachone series was chosen keeping in mind the biological properties played by the aryl-substituted lapachones **2** and **3**. However, in this work a cycloalkyl derivative, the cyclohexylthio-nor-β-lapachone **4g**, was also considered to see if the alkyl-type substituent would give any significative difference in the biological assessment.

Metalloporphyrins are recognized as being excellent biomimetic catalysts in reactions like hydroxylation, epoxidation, dehydrogenation, *N*-dealkylation, *N*-oxide formation or *S*-oxidation [33,34,35,36] and hydrogen peroxide is a cheap and environmentally-safe oxidant. Therefore, the work presented here is in consonance with our interests on structural modification of nor-β-lapachone derivatives and on the use of metalloporphyrins and hydrogen peroxide to obtain high value-added products [37,38,39,40,41,42,43]. Antibacterial activities of non-oxidized and oxidized lapachone thio-derivatives were assessed against Gram-positive (*Staphylococcus aureus* 2065 MA) and Gram-negative (*Escherichia coli* ATCC 13706) bacteria.

## 2. Results

### 2.1. Synthesis of Sulfonyl-nor-β-lapachone Derivatives ***7a**–**g***

**Scheme 1:**
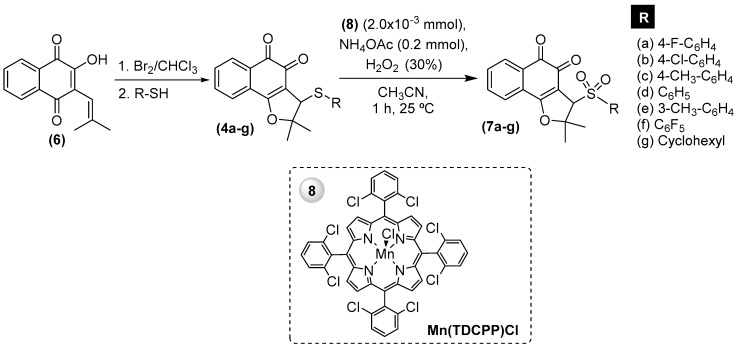
Synthetic access to naphthoquinones **4a**–**g** and the oxidation conditions used for their conversion to **7a**–**g**.

The synthetic strategy to obtain the new nor-β-lapachone derivatives **7a**–**g** involved the experimental work summarized in Scheme 1.

The starting nor-β-lapachone-3-thio-derivatives **4a**–**f** were obtained according to data in the literature [30] and the synthesis of **4g** is described here. The steps involved the reaction of nor-lapachol (**6**) with bromine, followed by the in situ quenching of the cyclic cationic *o*-quinone methide intermediate with the adequate thiol derivatives.

The oxidation of derivatives **4a**–**g** was performed by using the manganese (III) complex of 5,10,15,20-tetrakis(2,6-dichlorophenyl)porphyrin, Mn(TDCPP)Cl (**8**) as a catalyst and hydrogen peroxide as the oxidant (Scheme 1). All reactions were performed in acetonitrile at ambient temperature and the oxidant was added every 15 min to the reaction mixture in aliquots of 0.15 mmol. After 1 h of reaction, thin-layer chromatography (TLC) control showed the total or almost total consumption of each starting arylthio/cyclohexylthio-lapachones **4a**–**g** and the formation of a more polar product. After the workup and purification of the reaction mixture by preparative TLC using CH_2_Cl_2_ as eluent, followed by a detailed spectroscopic analysis of the major compound in each case, it was possible to identify the nor-β-lapachone sulfonyl-products **7a**–**g**, which were isolated in yields ranging from 78% to 86% (Table 1). The structures of all new derivatives were confirmed by ^1^H- and ^13^C-NMR, IR spectroscopy and high-resolution mass spectra (HRMS-ESI) (see Experimental Section and Supplementary Materials). In particular, the mass spectra of derivatives **7** show molecular ions containing 32 mass units higher than the corresponding molecular ions of their precursors **4**.

**Figure 3:**
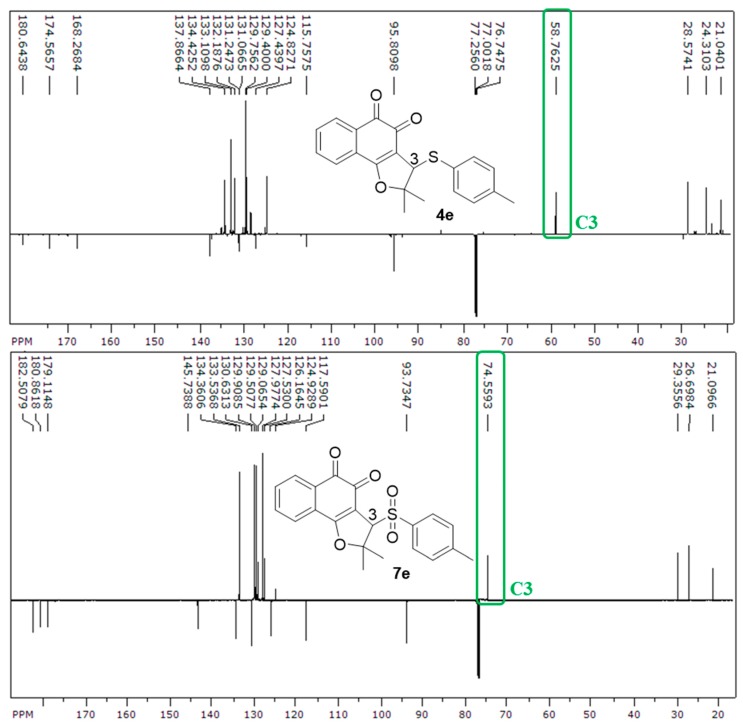
^13^C-Attached proton test (^13^C-APT) spectra of **4e** and **7e** in CDCl_3_.

Additionally, in the ^13^C-NMR spectrum the distinctive signal of C3 of each sulfone occurs at a higher chemical shift than the corresponding C3 of its precursor **4**, this being in agreement with the deprotection present in the product sulfone (Figure 3 shows the cases of **4e** and **7e**). None of the other signals were strongly affected.

### 2.2. Antibacterial Evaluation of Polyvinylpyrrolidone (PVP) Formulations of 3-Arylthio-nor-β-lapachone Derivatives ***4a**–**f***, 3-Cyclohexylthio-nor-β-lapachone ***4g*** and of the Corresponding Sulfonyl Nor-β-lapachone Derivatives ***7a**–**g***

Considering that sulfides and sulfones have already demonstrated antimicroorganism properties, the antibacterial activities of 3-arylthio/cyclohexylthio-nor-β-lapachones **4a**–**g** and corresponding oxidized nor-β-lapachone derivatives **7a**–**g** were evaluated against Gram-positive and Gram-negative bacteria. Since compounds **4** and **7** are not soluble in aqueous and/or physiological media, they were incorporated into polyvinylpyrrolidone micelles.

#### 2.2.1. Incorpo**r**ation of 3-Arylthio/Cyclohexylthio-nor-β-lapachone Derivatives **4a**–**g** and Nor-β-lapachone Derivatives **7a**–**g** into Polyvinylpyrrolidone Micelles

PVP is a water-soluble and non-toxic polymer, widely used to modify the water solubility of numerous biologically active compounds and also their pharmacokinetic and pharmacological activities [44]. In fact, PVP has been successfully applied in drug delivery of several antibiotics and antifungal agents, demonstrating high efficiency in the delivery of these drugs [45,46,47].

The micelles of PVP-arylthio/cyclohexylthio-lapachones **4a**–**g** and PVP-sulfonyl-lapachones **7a**–**g** were prepared by mixing chloroform solutions of PVP and lapachones (10:1 *w*/*w*). The resulting solutions were stirred for 2 h at room temperature and then the chloroform was evaporated under a nitrogen atmosphere. After this procedure, all residues were dissolved in 2 mL of water giving rise to the micelle solutions of PVP-arylthio/cyclohexylthio-lapachones **4a**–**g** and PVP-sulfonyl-lapachones **7a**–**g**.

#### 2.2.2. Antibacterial Evaluation of PVP Formulations of **4a**–**g** and **7a**–**g**

The PVP-arylthio/cyclohexylthio-lapachones **4a**–**g** and PVP-sulfonyl-lapachones’ **7a**–**g** micelles were screened for potential antimicrobial activity against Gram-positive bacteria (*Staphylococcus aureus* 2065 MA) and Gram-negative bacteria (*Escherichia coli* ATCC 13706), using the Kirby–Bauer disc diffusion method [48].

A standard concentration of both bacteria was plated on Mueller–Hinton agar (Liofilchem, Italy). The 6-mm discs (Liofilchem, Italy) were immersed in the solutions of the formulations PVP-arylthio/cyclohexylthio-lapachones **4a**–**g** and PVP-sulfonyl-lapachones **7a**–**g** at concentrations of 1 mM (concentration of each lapachone derivative) and placed on the plates that were incubated overnight at 37 °C. After incubation, the diameters of the inhibition halos were measured. In order to control the toxicity of PVP, a control experiment with a solution of PVP (10 mg/mL) was also carried out for each bacteria strain.

**Figure 4:**
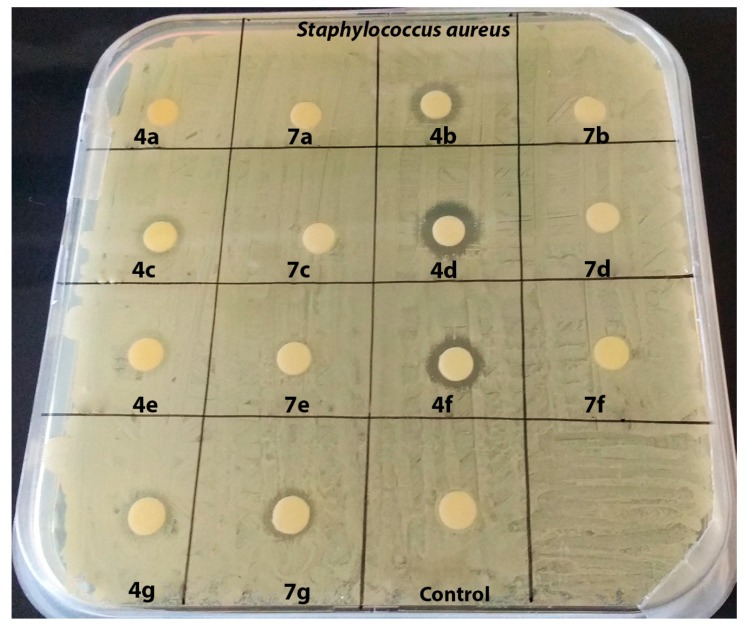
Picture of the plate containing *Staphylococcus aureus* plated on Mueller–Hinton agar and the discs immersed in the solutions of the formulations PVP-arylthio/cyclohexylthio-lapachones **4a**–**g** and PVP-sulfonyl-lapachones **7a**–**g** at concentrations of 1 mM (concentration of each lapachone derivative) after incubation overnight at 37 °C.

The results of the preliminary antimicrobial activity tests show that *E. coli* growth is not inhibited by any one of the lapachone derivatives **4a**–**g** and **7a**–**g**, since no inhibitory halos were formed. However, that was not the case with *S. aureus*; the inhibition halos’ features obtained are summarized in Table 2 and shown in Figure 4.

These results show that *S. aureus* presents sensitivity to arylthio/cyclohexylthio-lapachones **4b**–**g** and sulfonyl-lapachones’ **7e** and **7g** PVP formulations (Figure 4 and Table 2). It is important to emphasize that, with the exception of **4a**, all the other arylthio/cyclohexylthio-lapachones formulations were able to reduce the growth of *S. aureus*, with the larger inhibition halos obtained with **4d** and **4f**. However, with the sulfone derivatives, only compounds **7e** and **7g** have been demonstrated to maintain the anti-bacterial activity of *S. aureus*.

## 3. Experimental Section

### 3.1. Materials and Methods

A Mn(TDCPP)Cl (chloro [5,10,15,20-tetrakis-(2,6-dichlorophenyl)-porphyrinato] manganese(III)) catalyst was prepared by a procedure previously described in literature [42]. The 3-arylthio/cyclohexylthio-nor-β-lapachone derivatives **4a**–**g** were prepared according to a procedure described in the literature [30]. Other reagents and solvents used in the experimental work were purchased from Sigma-Aldrich (Jurubatuba, Brazil). The 3-sulfonyl-nor-β-lapachone derivatives **7a**–**g** were characterized by spectroscopic techniques such as ^1^H- and ^13^C-APT nuclear magnetic resonance, infrared (FT-IR) and mass spectrometry.

Melting points were obtained on a Fischer Jones apparatus and are uncorrected. Analytical grade solvents were used. Reagents were purchased from Aldrich. Column chromatography was performed on silica gel 60 (Merck 230–400 mesh, Rio de Janeiro, Brazil). Yields refer to purified compounds obtained by chromatographic techniques and confirmed by spectroscopic data. Reactions were monitored by thin-layer chromatography (TLC) performed on 0.25-mm E. Merck silica gel plates (60F-254) using UV light as the visualizing agent. Infrared spectra were recorded on a Perkin–Elmer FT-IR Spectrum One spectrophotometer (Jardim das Laranjeiras, Brazil), calibrated relatively to the 1601.8 cm^−1^ absorbance of polystyrene. NMR spectra were recorded on a Varian Unity Plus VXR (500 MHz) instrument in DMSO-*d*_6_ or CDCl_3_ solutions. The chemical shift data are reported in units of δ (ppm) downfield from tetramethylsilane or the solvent, either of which were used as the internal standard.

Coupling constants (*J*) are reported in Hertz and refer to apparent peak multiplicities. High-resolution mass spectra (HRMS) were recorded on a mass spectrometer, MICROMASS Q-TOF (Waters, Barueri, Brazil).

### 3.2. Synthesis of 3-Cyclohexylthio-nor-β-lapachone *(**4g**)*

A round-bottom flask containing a solution of nor-lapachol (500 mg, 2.2 mmol) in 40 mL of dry chloroform was externally cooled with ice and under an inert atmosphere, 4.4 mL (13.2 g, 8.36 mol) of bromine was added. Immediately a red solid precipitate of the cationic *ortho*-quinone methide derivative was formed; the reaction mixture was left stirring for additional 10 min. The bromine excess was removed under reduced pressure and immediately chloroform (40 mL) was added; the mixture was kept externally cool in an ice bath. To that mixture, chloroform solution with cyclohexanethiol (511 mg, 4.4 mmol in 25 mL) was slowly added. The reaction mixture was stirred for another 3 h and then poured into distilled water (50 mL). The organic phase was collected and successively washed with aqueous sodium bicarbonate solution (3 × 50 mL), and distilled water (3 × 50 mL), dried over anhydrous sodium sulfate, filtered and evaporated under reduced pressure. The resulting red solid was taken in chloroform and was purified by silica gel flash column chromatography, eluting with gradient mixtures of hexane and ethyl acetate.

*3-(Cyclohexylthio)-2,2-dimethyl-2,3-dihydronaphtho[1,2-b]furan-4,5-dione* (**4g**). Compound **4g** was isolated as an orange solid in 81% yield. m.p. 155 °C; IR (KBr, cm^−1^): ν 2931, 2849, 1654, 1644, 1614, 1587, 1569, 1449, 1399, 1248, 1219, 1079; ^1^H-NMR (CDCl_3_, 500 MHz): δ 1.20–1.40 (6H, m, H-3’, H-4’, H-5’), 1.56 (3H, s, (C-2)-CH_3_), 1.71 (3H, s, (C-2)-CH_3_), 1.74–1.80 (2H, m, H-2’or H-6’), 1.96–2.11 (2H, m, H-2’or H-6’), 3.06–3.15 (1H, m, H1’), 4.16 (1H, s, H-3), 7.55–7.65 (1H, m, H-7), 7.55–7.65 (1H, m, H-8), 7.55–7.65 (1H, m, H-9), 8.08 (1H, dt, *J* = 7.3 and 1.1 Hz, H-6); ^13^C-NMR (CDCl_3_, 125 MHz): δ 24.4 (C-2)-CH_3_), 25.7 (C-4’), 25.9 (C-3’ or C-5’), 26.1 (C-3’ or C-5’), 28.4 (C-2)-CH_3_), 33.4 (C-2’ or C-6’), 34.5 (C-2’ or C-6’), 45.1 (C-1’), 52.2 (C-3), 95.2 (C-2), 118.3 (C-3a), 127.7 (C-9a), 129.3 (C-6), 130.9 (C-5a), 124.7, 132.0 and 134.4 (C-7, C-8 and C-9), 167.8 (C-9b), 175.0 (C-4), 180.9 (C-5). Anal. Calcd. for C_20_H_22_O_3_S (342.45 g/mol): 69.19% C; 6.38% H. Found: 69.14 % C; 6.37% H. HRMS: exact mass calculated for C_20_H_22_O_3_SNa^+^ 365.1182. Found 365.1193.

### 3.3. General Procedure for the Synthesis of 3-Aryl/Cyclohexyl-sulfonyl-nor-β-lapachone Derivatives *(**7a**–**g**)*

For the catalytic studies, each substrate of **4a**–**g** (0.3 mmol), the catalyst (2.0 × 10^−3^ mmol, where the sub/cat molar ratio used was 150) and the co-catalyst (ammonium acetate, 0.2 mmol) were dissolved in CH_3_CN (2.0 mL). The reaction mixtures were kept under magnetic stirring and in the absence of light at 22–25 °C. The oxidant, 30% H_2_O_2_ (*w*/*w*, aqueous solution), was diluted with CH_3_CN (1:10) and this was followed by the addition of 0.15 mmol of the oxidant taking place at every 15 min. The reactions were followed by TLC. For all the substrates, the resulting sulfones were isolated by preparative TLC using CH_2_Cl_2_ as eluent.

*3-(4-Fluorophenyl-sulfonyl)-2,2-dimethyl-2,3-dihydronaphtho[1,2-b]furan-4,5-dione* (**7a**). Compound **7a** was isolated as an orange solid in 84% yield. m.p. 188–190 °C; IR (KBr, cm^−1^): ν 1656, 1639, 1619, 1584, 1573, 1500, 1402, 1218, 1160, 829; ^1^H-NMR (CDCl_3_, 500 MHz): δ 1.16 (3H, s, (C-2)-CH_3_), 2.27 (3H, s, (C-2)-CH_3_), 4.58 (1H, s, H-3), 7.21 (2H, t, *J* = 8.5 Hz, H-2’ and H-6’), 7.65–7.73 (2H, m, H-3’ and H-5’), 7.76–7.86 (1H, m, H-7), 7.76–7.86 (1H, m, H-8), 7.76–7.86 (1H, m, H-9), 8.10 (1H, d, *J* = 7.2 Hz, H-6); ^13^C-NMR (CDCl_3_, 125 MHz): δ 22.7 (C-2)-CH_3_), 29.7 (C-2)-CH_3_), 71.9 (C-3), 95.6 (C-2), 110.7 (C-3a), 116.6 (C-3’), 116.9 (C-5’), 125.7 (C-9a), 129.7 (C-6), 131.9 (C-1’), 132.0 (C-5a), 133.3 (C-6’), 134.97 (C-2’), 125.7, 132.0 and 134.9 (C-7, C-8 and C-9), 170.5 (C-4’), 174.4 (C-9b), 176.8 (C-4), 180.2 (C-5). HRMS: exact mass calculated for C_20_H_15_FO_5_SNa^+^ 409.0522. Found 409.0523. HRMS: exact mass calculated for C_20_H_15_FO_5_SH^+^ 387.0702. Found 387.0700.

*3-(4-Chlorophenyl-sulfonyl)-2,2-dimethyl-2,3-dihydronaphtho[1,2-b]furan-4,5-dione* (**7b**). Compound **7b** was isolated as an orange solid in 86% yield. m.p. 226–228 °C; IR (KBr, cm^−1^): ν 2926, 1660, 1644, 1615, 1567, 1477, 1403, 1251, 1220, 1095, 1004, 790; ^1^H-NMR (CDCl_3_, 500 MHz): δ 1.54 (3H, s, (C-2)-CH_3_), 2.10 (3H, s, (C-2)-CH_3_), 4.87 (1H, s, H-3), 7.17 (2H, dd, *J* = 7.8 and 1.9 Hz, H-2’ and H-6’), 7.34 (2H, dd, *J* = 8.3 and 1.9 Hz, H-3’ and H-5’), 7.47–7.63 (1H, m, H-7), 7.47–7.63 (1H, m, H-8), 7.47–7.63 (1H, m, H-9), 7.82 (1H, d, *J* = 7.3 Hz, H-6); ^13^C-NMR (CDCl_3_, 125 MHz): δ 22.9 (C-2)-CH_3_), 29.7 (C-2)-CH_3_), 71.8 (C-3), 95.6 (C-2), 110.4 (C-3a), 126.8 (C-9a), 128.2, 128.8 (C-2’ and C-6’), 129.2 (C-6), 131.0 (C-5a), 132.0 (C-4’), 133.6 (C-3’ and C-5’), 134.5 (C-1’), 125.6, 132.6 and 134.8 (C-7, C-8 and C-9), 170.0 (C-9b), 174.8 (C-4), 180.5 (C-5). HRMS: exact mass calculated for C_20_H_15_ClO_5_SNa^+^ 425.0226. Found 425.0225. HRMS: exact mass calculated for C_20_H_15_ClO_5_SH^+^ 368.0718. Found 368.0717.

*2,2-Dimethyl-3-tosylsulfonil-2,3-dihydronaphtho[1,2-b]furan-4,5-dione* (**7c**). Compound **7c** was isolated as an orange solid in 81% yield. m.p. 185–188 °C; IR (KBr, cm^−1^): ν 1653, 1641, 1611, 1590, 1563, 1490, 1405, 1224, 1077, 788; ^1^H-NMR (CDCl_3_, 500 MHz): δ 1.63 (3H, s, (C-2)-CH_3_), 1.84 (3H, s, (C-2)-CH_3_), 2.04 (3H, s, (C-4’)-CH_3_), 4.52 (1H, s, H-3), 6.76 (2H, dd, *J* = 8.1 and 2.0 Hz, H-3’ and H-5’), 7.49 (2H, dd, *J* = 8.0 and 2.1 Hz, H-2’ and H-6’), 7.56–7.65 (1H, m, H-7), 7.56–7.65 (1H, m, H-8), 7.56–7.65 (1H, m, H-9), 8.05 (1H, d, *J* = 7.8 Hz, H-6); ^13^C-NMR (CDCl_3_, 125 MHz): δ 21.1 (C-2)-CH_3_), 27.6 (C-2)-CH_3_), 30.4 (C-4’)-CH_3_), 74.7 (C-3), 96.0 (C-2), 118.9 (C-3a), 126.5 (C-9a), 128.1, 128.6 (C-2’ and C-6’), 129.8 (C-6), 131.1 (C-5a), 131.4 (C-1’), 133.3 (C-3’ and C-5’), 125.6, 133.2 and 134.7 (C-7, C-8 and C-9), 147.5 (C-4’), 171.1 (C-9b), 174.4 (C-4), 180.0 (C-5). HRMS: exact mass calculated for C_21_H_18_O_5_SNa^+^ 405.0773. Found 405.0771. HRMS: exact mass calculated for C_21_H_18_O_5_SH^+^ 383.0953. Found 383.0954.

*2,2-Dimethyl-3-(phenylsulfonyl)-2,3-dihydronaphtho[1,2-b]furan-4,5-dione* (**7d**). Compound **7d** was isolated as an orange solid in 85% yield. m.p. 231–233 °C; IR (KBr, cm^−1^): ν 1653, 1640, 1615, 1585, 1570, 1400, 1243, 1222, 1078; ^1^H-NMR (CDCl_3_, 500 MHz): δ 1.62 (3H, s, (C-2)-CH_3_), 1.89 (3H, s, (C-2)-CH_3_), 4.87 (1H, s, H-3), 7.41 (3H, t, *J* = 7.8 Hz, H-3’, H-4’, H-5’), 7.5–7.59 (2H, m, H-2’, H-6’), 7.65–7.82 (1H, m, H-7), 7.65–7.82 (1H, m, H-8), 7.65–7.82 (1H, m, H-9), 7.97 (1H, dd, *J* = 7.8, 1.4 Hz, H-6); ^13^C-NMR (CDCl_3_, 125 MHz): δ 23.2 (C-2)-CH_3_), 30.7 (C-2)-CH_3_), 73.9 (C-3), 100.6 (C-2), 110.8 (C-3a), 127.6 (C-4’), 128.2 (C-3’, C-5’), 128.7 (C-9a), 129.7 (C-6), 130.8 (C-5a), 133.8 (C-2’, C-6’), 125.0, 132.8, 134.8 (C-7, C-8, C-9), 134.6 (C-1’), 170.5 (C-9b), 189.5 (C-4), 192.3 (C-5). HRMS: exact mass calculated for C_20_H_16_O_5_SNa^+^ 391.0616. Found 391.0617. HRMS: exact mass calculated for C_20_H_16_O_5_SH^+^ 369.0797. Found 360.0798.

*2,2-Dimethyl-3-(m-tolylsulfonyl)-2,3-dihydronaphtho[1,2-b]furan-4,5-dione* (**7e**). Compound **7e** was isolated as an orange solid in 80% yield. m.p. 240–242 °C; IR (KBr, cm^−1^): ν 1654, 1643, 1620, 1588, 1572, 1400, 1243, 1220, 1081, 772; ^1^H-NMR (CDCl_3_, 500 MHz): δ 1.46 (3H, s, (C-2)-CH_3_), 1.53 (3H, s, (C-2)-CH_3_), 1.92 (3H, s, (C-3’)-CH_3_), 4.67 (1H, s, H-3), 7.36 (1H, t, *J* = 7.8 Hz, H-4’), 7.50–7.59 (1H, m, H-7), 7.50–7.59 (1H, m, H-8), 7.50–7.59 (1H, m, H-9), 7.66–7.68 (2H, m, H-5’ and H-6’), 7.84 (1H, d, *J* = 7.7 Hz, H-2’), 7.93 (1H, d, *J* = 7.8 Hz, H-6); ^13^C-NMR (CDCl_3_, 125 MHz): δ 21.8 (C-2)-CH_3_), 25.3 (C-2)-CH_3_), 29.8 (C-3’)-CH_3_), 74.5 (C-3), 94.3 (C-2), 118.1 (C-3a), 126.7 (C-9a), 128.0 (C-6’), 128.5 (C-6), 129,6 (C-2’), 130.4 (C-4’), 131.2 (C-5a), 133.5 (C-5’), 134.9 (C-1’), 125.4, 130.4 and 134.0 (C-7, C-8 and C-9), 144.0 (C-3’), 170.8 (C-9b), 179.6 (C-4), 181.4 (C-5). HRMS: exact mass calculated for C_21_H_18_O_5_SNa^+^ 405.0773. Found 405.0772. HRMS: exact mass calculated for C_21_H_18_O_5_SH^+^ 383.0953. Found 383.0954.

*3-(Pentafluorophenylsulfonyl)-2,2-dimethyl-2,3-dihydronaphtho[1,2-b]furan-4,5-dione* (**7f**). Compound **7f** was isolated as an orange solid in 78% yield. m.p. 235–237 °C; IR (KBr, cm^−1^): ν 1660, 1643, 1622, 1574, 1513, 1490, 1402, 1219, 1087, 978, 853; ^1^H-NMR (CDCl_3_, 500 MHz): δ 1.60 (3H, s, (C-2)-CH_3_), 1.98 (3H, s, (C-2)-CH_3_), 5.29 (1H, s, H-3), 7.40–7.59 (1H, m, H-7), 7.40–7.59 (1H, m, H-8), 7.40–7.59 (1H, m, H-9), 8.07 (1H, d, *J* = 7.3 Hz, H-6); ^13^C-NMR (CDCl_3_, 125 MHz): δ 22.7 (C-2)-CH_3_), 29.9 (C-2)-CH_3_), 74.7 (C-3), 96.6 (C-2), 111.8 (C-3a), 120.7 (C-1’), 128.5(C-9a), 129.3 (C-6), 130.4 (C-5a), 124.2, 131.3 and 134.9 (C-7, C-8 and C-9), 137.5 (C-4’), 144.5 (C-3’ and C-5’), 148.31 (C-2’ and C-6’), 170.5 (C-9b), 175.9 (C-4), 180.0 (C-5). HRMS: exact mass calculated for C_20_H_11_F_5_O_5_SNa^+^ 481.0145. Found 481.0146. HRMS: exact mass calculated for C_20_H_11_F_5_O_5_SH^+^ 459.0326. Found 459.0325.

*3-(Cyclohexylsulfonyl)-2,2-dimethyl-2,3-dihydronaphtho[1,2-b]furan-4,5-dione* (**7g**). Compound **7g** was isolated as an orange solid in 81% yield. m.p. 222–225 °C; IR (KBr, cm^−1^): ν 2931, 2849, 1654, 1644, 1614, 1587, 1569, 1449, 1399, 1248, 1219, 1079; ^1^H-NMR (CDCl_3_, 500 MHz): δ 1.20–1.40 (6H, m, H-3’, H-4’, H-5’), 1.56 (3H, s, (C-2)-CH_3_), 1.71 (3H, s, (C-2)-CH_3_), 1.74–1.80 (2H, m, H-2’or H-6’), 1.96–2.11 (2H, m, H-2’or H-6’), 3.06–3.15 (1H, m, H1’), 4.16 (1H, s, H-3), 7.55–7.65 (1H, m, H-7), 7.55–7.65 (1H, m, H-8), 7.55–7.65 (1H, m, H-9), 8.08 (1H, dt, *J* = 7.3 and 1.1 Hz, H-6); ^13^C-NMR (CDCl_3_, 125 MHz): δ 24.4 (C-2)-CH_3_), 25.7 (C-4’), 25.9 (C-3’ or C-5’), 26.1 (C-3’ or C-5’), 28.4 (C-2)-CH_3_), 33.4 (C-2’ or C-6’), 34.5 (C-2’ or C-6’), 45.1 (C-1’), 73.4 (C-3), 95.2 (C-2), 118.3 (C-3a), 127.7 (C-9a), 129.3 (C-6), 130.9 (C-5a), 124.7, 132.0 and 134.4 (C-7, C-8 and C-9), 167.8 (C-9b), 175.0 (C-4), 180.9 (C-5). HRMS: exact mass calculated for C_20_H_22_O_5_SNa^+^ 397.1086. Found 397.1088. HRMS: exact mass calculated for C_20_H_22_O_5_SH^+^ 375.1266. Found 375.1264.

### 3.4. General Procedure for the Incorporation of 3-Arylthio/cyclohexylthio-nor-β-lapachone Derivatives ***4a**–**g*** and 3-Aryl/Cyclohexyl-sulfonyl-nor-β-lapachone ***7a**–**g*** Derivatives into PVP Micelles

To chloroform solutions of PVP (20 mg in 2 mL), a chloroform solution of ca. 2 mg of each 3-arylthio/cyclohexylthio-nor-β-lapachones **4a**–**g** or 3-aryl/cyclohexyl-sulfonyl-nor-β-lapachone **7a**–**g** (in 2 mL of chloroform) was added. The resulting solutions were stirred for 2 h at room temperature and then the chloroform was evaporated under nitrogen atmosphere. In order to remove all the organic solvents, the residues were kept in an oven at 45 °C for 24 h. After this procedure, all residues were dissolved in 2 mL of water, leading to the aqueous solution of PVP-arylthio/cyclohexylthio-lapachones **4a**–**g** and PVP-sulfonyl-lapachones **7a**–**g** micelles.

### 3.5. Antibacterial Evaluation of PVP Formulations of 3-Arylthio/Cyclohexylthio-nor-β-lapachone Derivatives ***4a**–**g*** and 3-sulfonyl-nor-β-lapachones ***7a**–**g***

*Staphylococcus aureus* 2065 MA and *Escherichia coli* ATCC 13706 from fresh cultured plates were inoculated in tryptic soy broth (TSB) and grown overnight aerobically at 37 °C under 100 rpm. Then, an aliquot was transferred into fresh TSB at the same growth conditions to reach the early stationary phase. For *E. coli*, an optical density at 600 nm (OD_600_) of 1.6 ± 0.1 corresponded to ∼10^8^ colony forming units (CFU)·mL^−1^. For *S. aureus*, an OD_600_ of 1.9 ± 0.1 corresponded to ∼10^8^ CFU·mL^−1^.

The antibacterial evaluation of PVP formulations of 3-arylthio/cyclohexylthio-nor-β-lapachone derivatives **4a**–**g** and 3-sulphonyl-nor-β-lapachones **7a**–**g** was done according to the European Committee on Antimicrobial Susceptibility Testing standards (EUCAST 2015). The bacterial cultures of *S. aureus* and *E. coli* cultivated in TSB were diluted 1:100 in 0.85% saline solution to obtain a density of 0.5 MacFarland. After that, for each bacterium a sterile cotton swab was dipped into the suspension and the inoculum was spread over the entire surface of a Muller–Hinton plate by swabbing in three directions. Then, sterilized disks were immersed into the compounds with a concentration of 1 mM and placed at the plate and incubated inverted at 37 °C for 16–20 h. The diameters of inhibition zones were measured.

## 4. Conclusions

Herein we present a new, efficient and environmentally friendly methodology, involving the use of a manganese (III) porphyrin complex as a catalyst and aqueous hydrogen peroxide as an oxidant in order to promote the oxidation of several organosulfur derivatives of nor-β-lapachone. This methodology allowed the preparation of the new sulfonyl-lapachones **7a**–**g** in excellent yields through the oxidation of arylthio/cyclohexylthio-lapachones **4a**–**g**.

For the antibacterial activity studies, the lapachone derivatives were successfully incorporated in polyvinylpyrrolidone (PVP) micelles. The PVP-arylthio/cyclohexylthio-lapachones and PVP-sulfonyl-lapachones’ micelles were tested against a Gram-positive (*S. aureus*) and a Gram-negative (*E. coli*) bacteria. The preliminary results showed that such formulations are not active against *E. coli*. However, the PVP formulations with arylthio/cyclohexylthio-lapachones **4b**–**g** and with sulfonyl-lapachones **7e** and **7g** reduced the growth of *S. aureus.* These compounds can be considered as prototypes for future antibacterial agents.

## Figures and Tables

**Table 1:** Results obtained in the oxidation reactions of **4a**–**g** to **7a**–**g**.

Entry	Compounds 4	η, Compounds 7 (%)
1	**a**	84
2	**b**	86
3	**c**	81
4	**d**	85
5	**e**	80
6	**f**	78
7	**g**	81

**Table 2:** Growth inhibition halo diameters (mm) for the polyvinylpyrrolidone (PVP)-arylthio/cyclohexylthio-lapachones **4a**–**g** and PVP-sulfonyl-lapachones **7a**–**g** (1 mM concentration of each lapachone derivative) against *Staphylococcus aureus*.

PVP Formulation (1 mM)	4a	4b	4c	4d	4e	4f	4g	7a	7b	7c	7d	7e	7f	7g
Gram-positive bacteria *S. aureus* 2065 MA	0	9	8	11	8	10	8	0	0	0	0	7	0	8

## Supplementary Materials

Supplementary materials are available online.
