# Supplementary material for: Efficient Catalytic Oxidation of 3-Arylthio- and 3-Cyclohexylthio-lapachone Derivatives to New Sulfonyl Derivatives and Evaluation of Their Antibacterial Activities
Source: Molecules. 2017 Feb 16;22(2):302. doi: 10.3390/molecules22020302 (PMC6155948; doi:10.3390/molecules22020302)
Supplement: Supplementary file 1 [file molecules-22-00302-s001.pdf]

Supplementary Materials

Efficient Catalytic Oxidation of 3-Arylthio- and 3-Cyclohexylthio-lapachone Derivatives to New Sulfonyl Derivatives and Evaluation of Their Antibacterial Activities

Mariana F. do C. Cardoso, Ana T. P. C. Gomes, Caroline dos S. Moreira, Mário M. Q. Simões, Maria G. P. M. S. Neves, David R. da Rocha, Fernando de C. da Silva, Catarina Moreirinha, Adelaide Almeida, Vitor F. Ferreira and José A. S. Cavaleiro

Figure 01: NMR and mass spectral data of the compound **7a** ---------------Pages 2 and 3

Figure 02: NMR and mass spectral data of the compound **7b** ---------------Pages 4 and 5

Figure 03: NMR and mass spectral data of the compound **7c** ---------------Pages 6 and 7

Figure 04: NMR and mass spectral data of the compound **7d** ---------------Pages 8 and 9

Figure 05: NMR and mass spectral data of the compound **7e** -------------Pages10 and 11

Figure 06: NMR and mass spectral data of the compound **7f** -------------Pages 12 and 13

Figure 07: NMR and mass spectral data of the compound **7g** ------------Pages 14 and 15

Figure 08: NMR and mass spectral data of the compound **4g** --------Pages 16, 17 and 18

3-(4-fluorophenyl)sulfonyl-2,2-dimethyl-2,3-dihydronaphtho[1,2-b]furan-4,5-dione **(7a)**

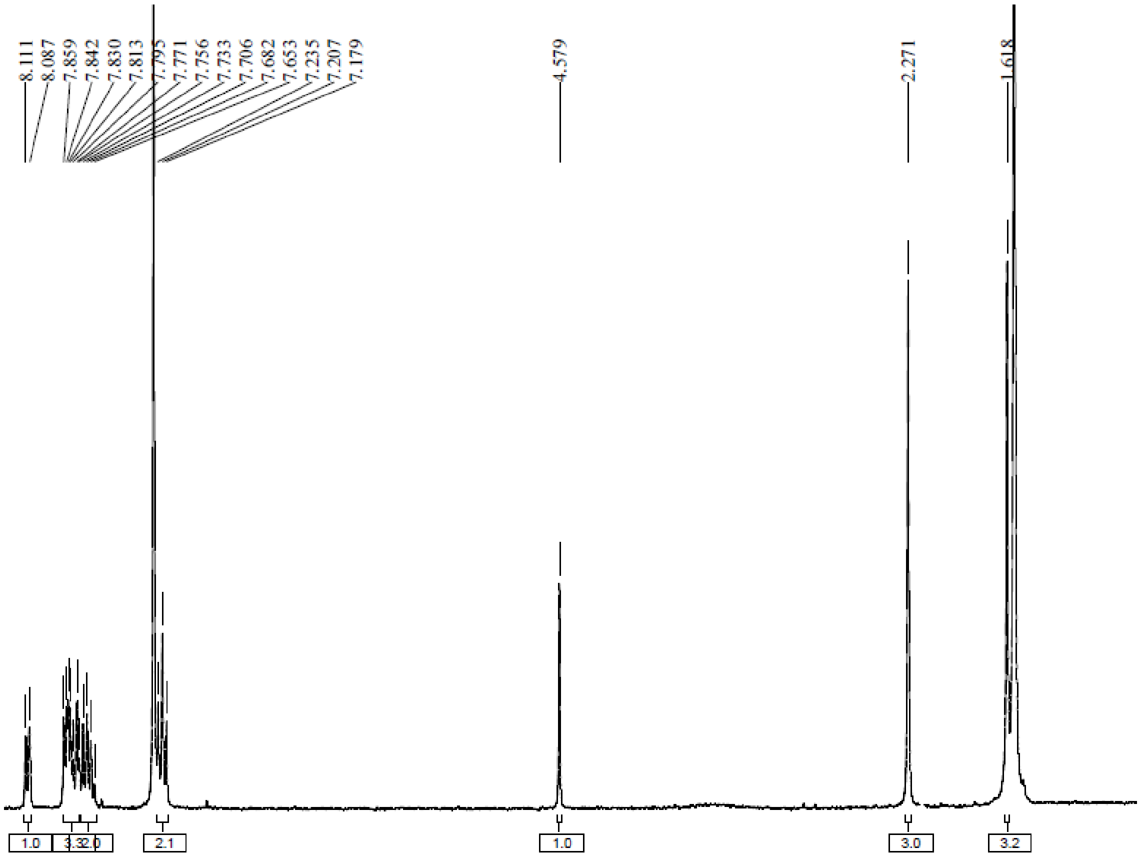

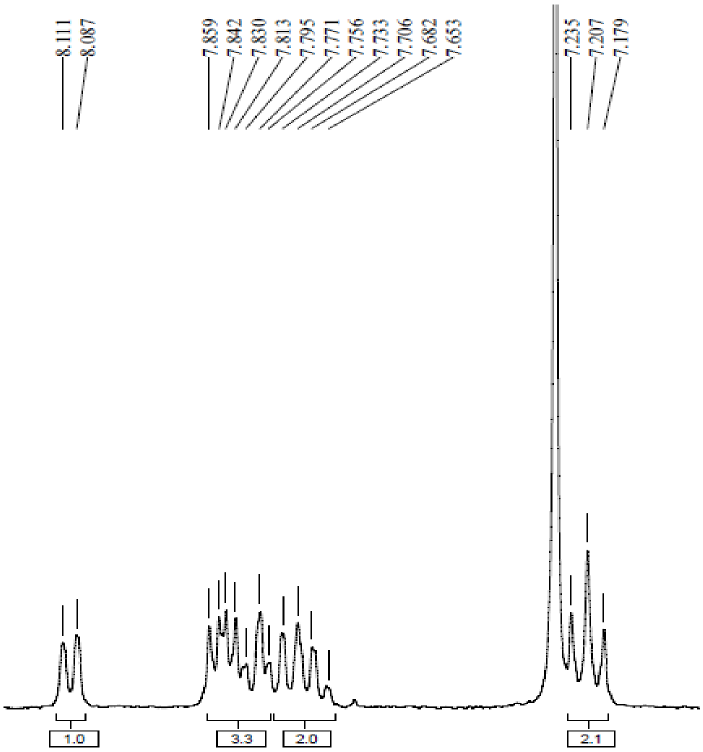

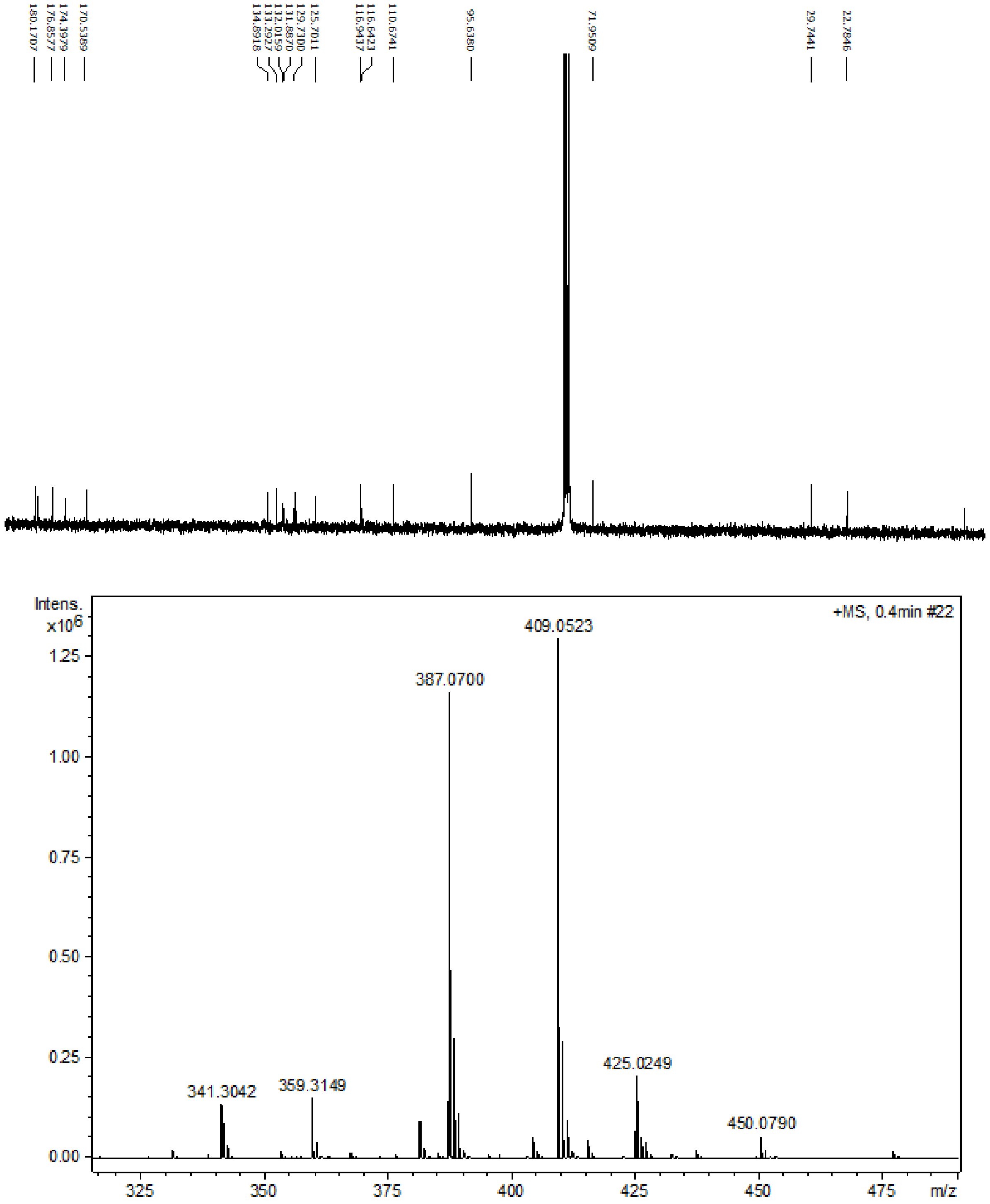

Figure 01: NMR and mass spectral data of the compound **7a**.

3-(4-chlorophenyl)sulfonyl-2,2-dimethyl-2,3-dihydronaphtho[1,2-b]furan-4,5-dione

(7b)

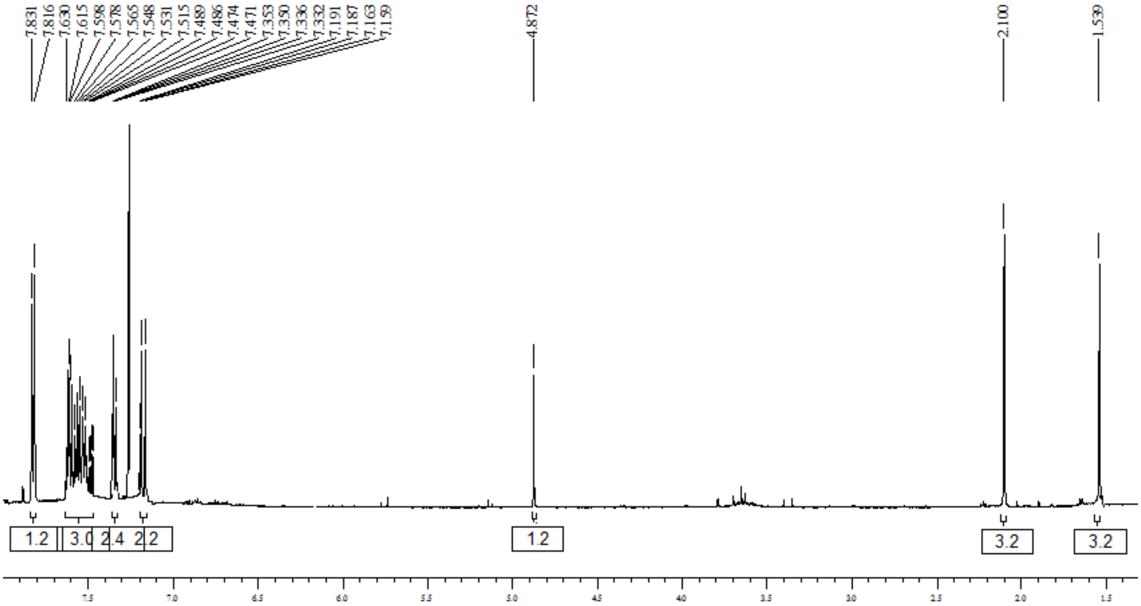

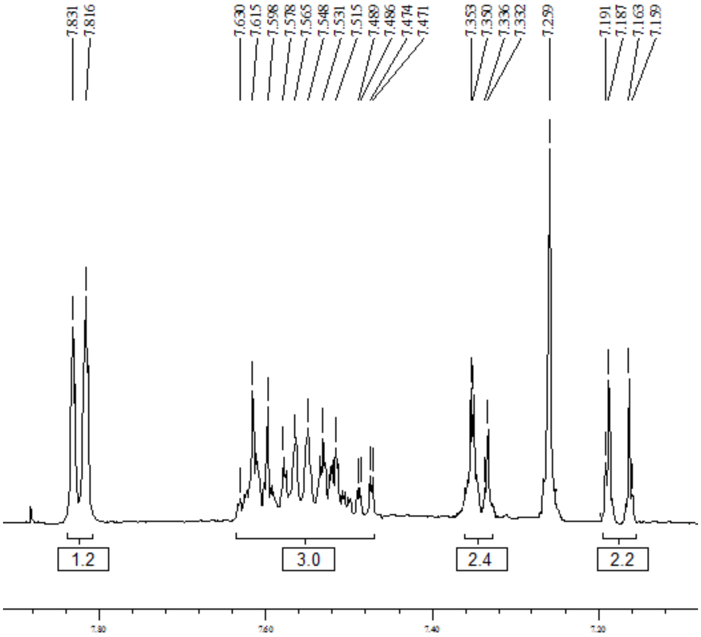

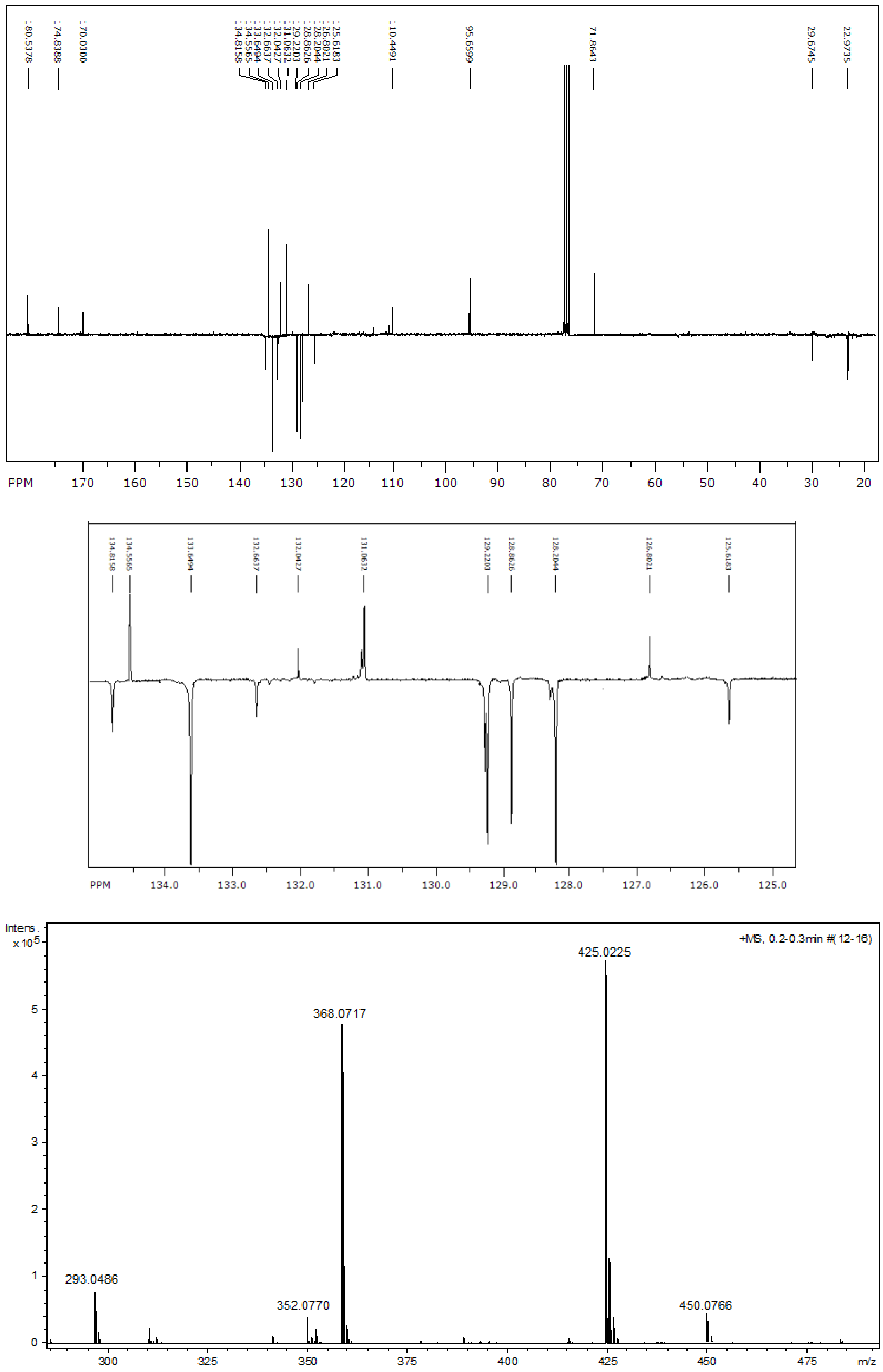

Figure 02: NMR and mass spectral data of the compound **7b**

2,2-dimethyl-3-tosylsulfonil-2,3-dihydronaphtho[1,2-b]furan-4,5-dione **(7c)**

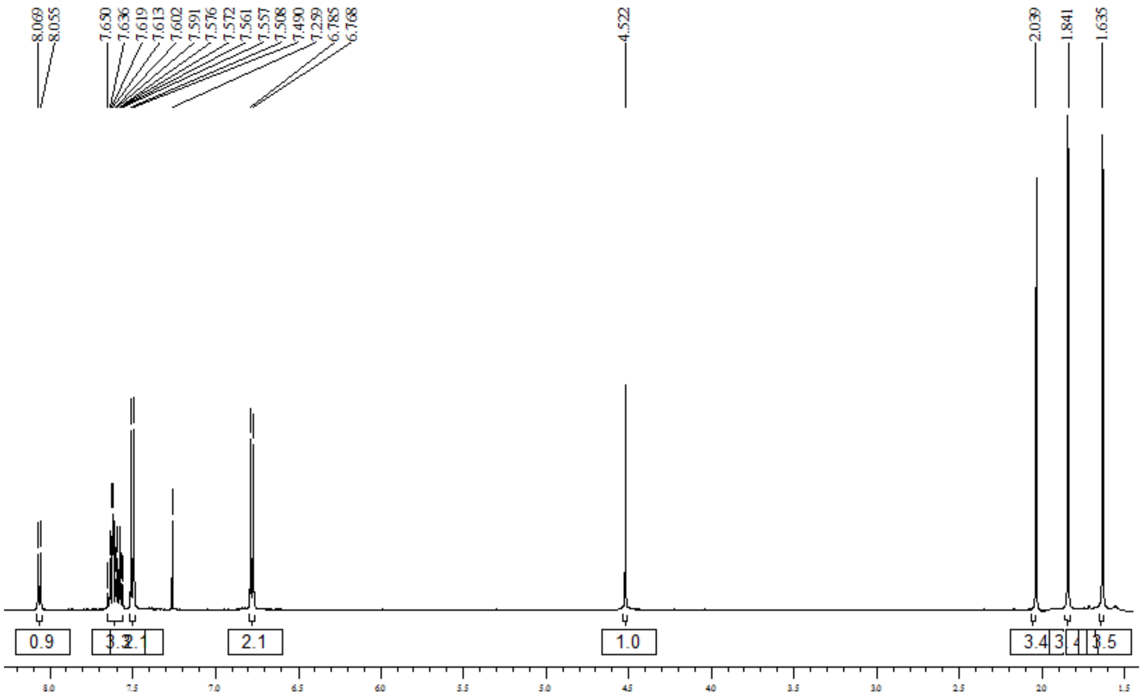

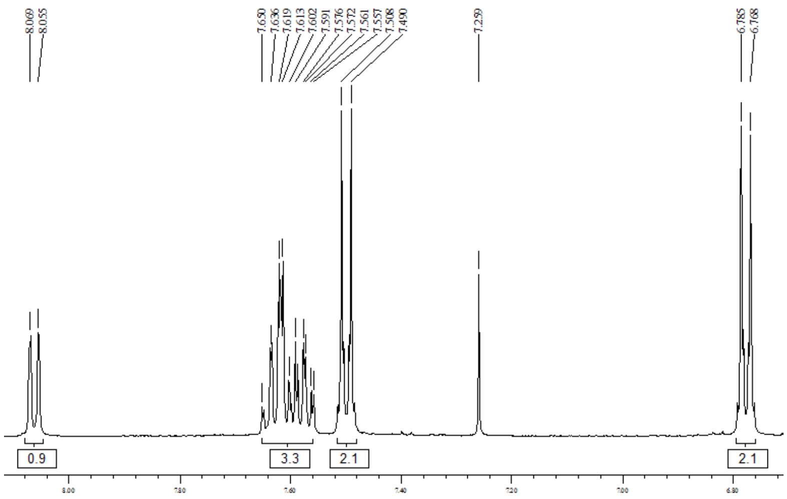

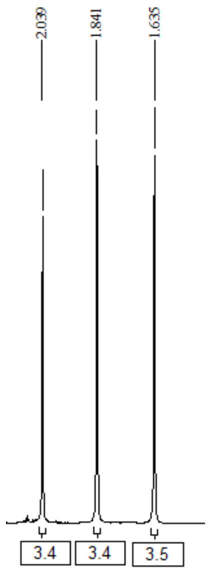

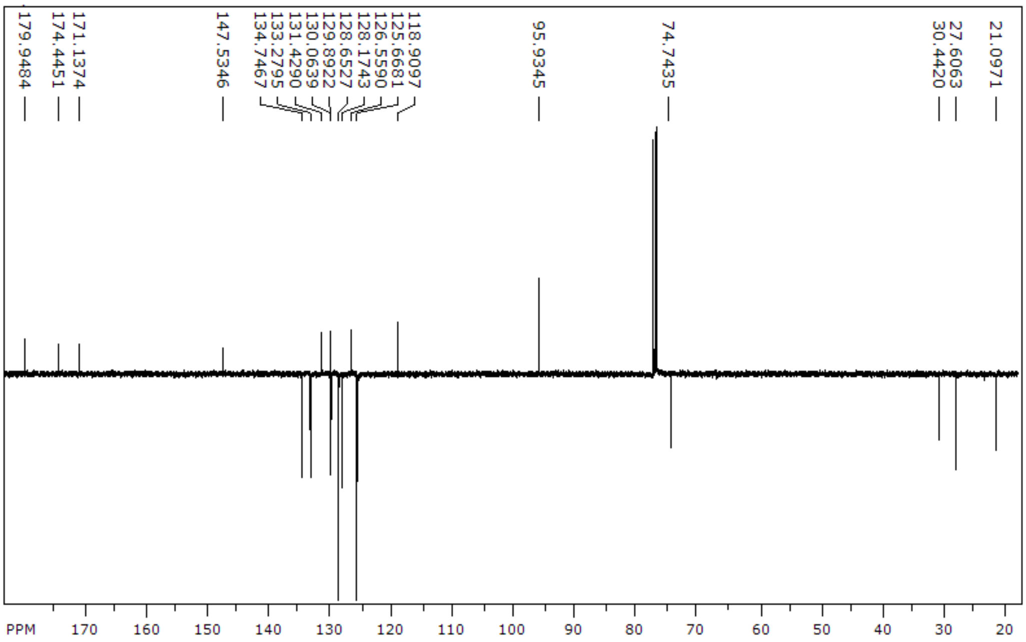

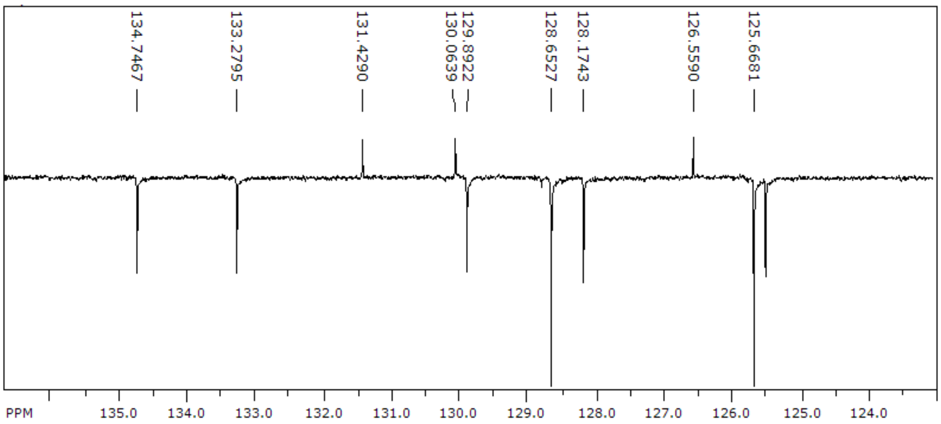

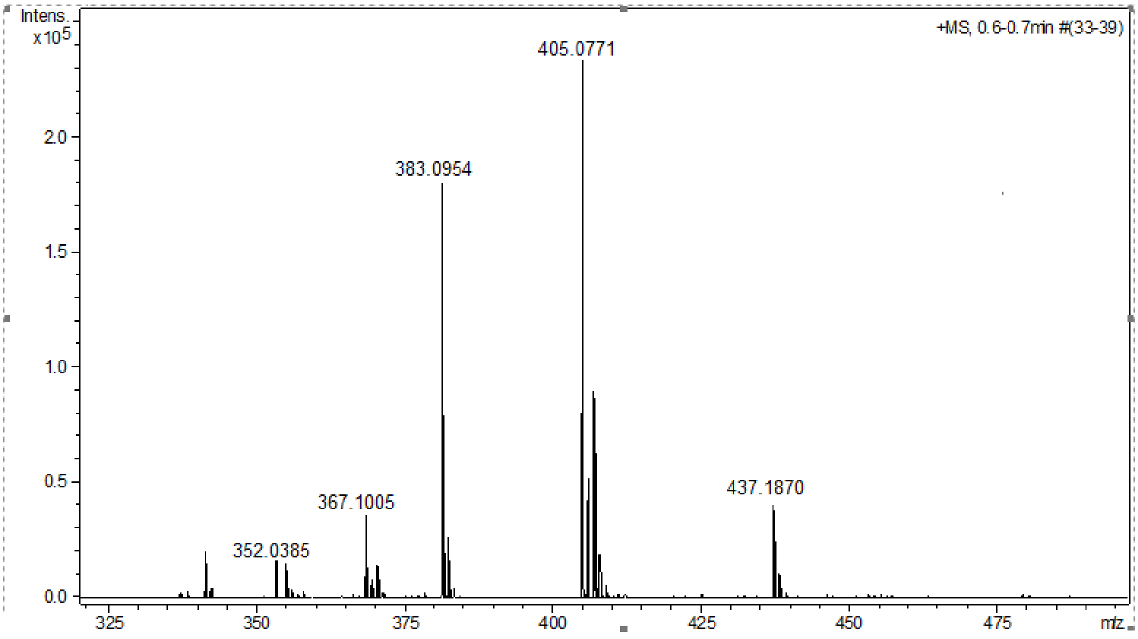

Figure 03: NMR and mass spectral data of the compound **7c**

2,2-dimethyl-3-phenylsulfonyl-2,3-dihydronaphtho[1,2-b]furan-4,5-dione **(7d)**

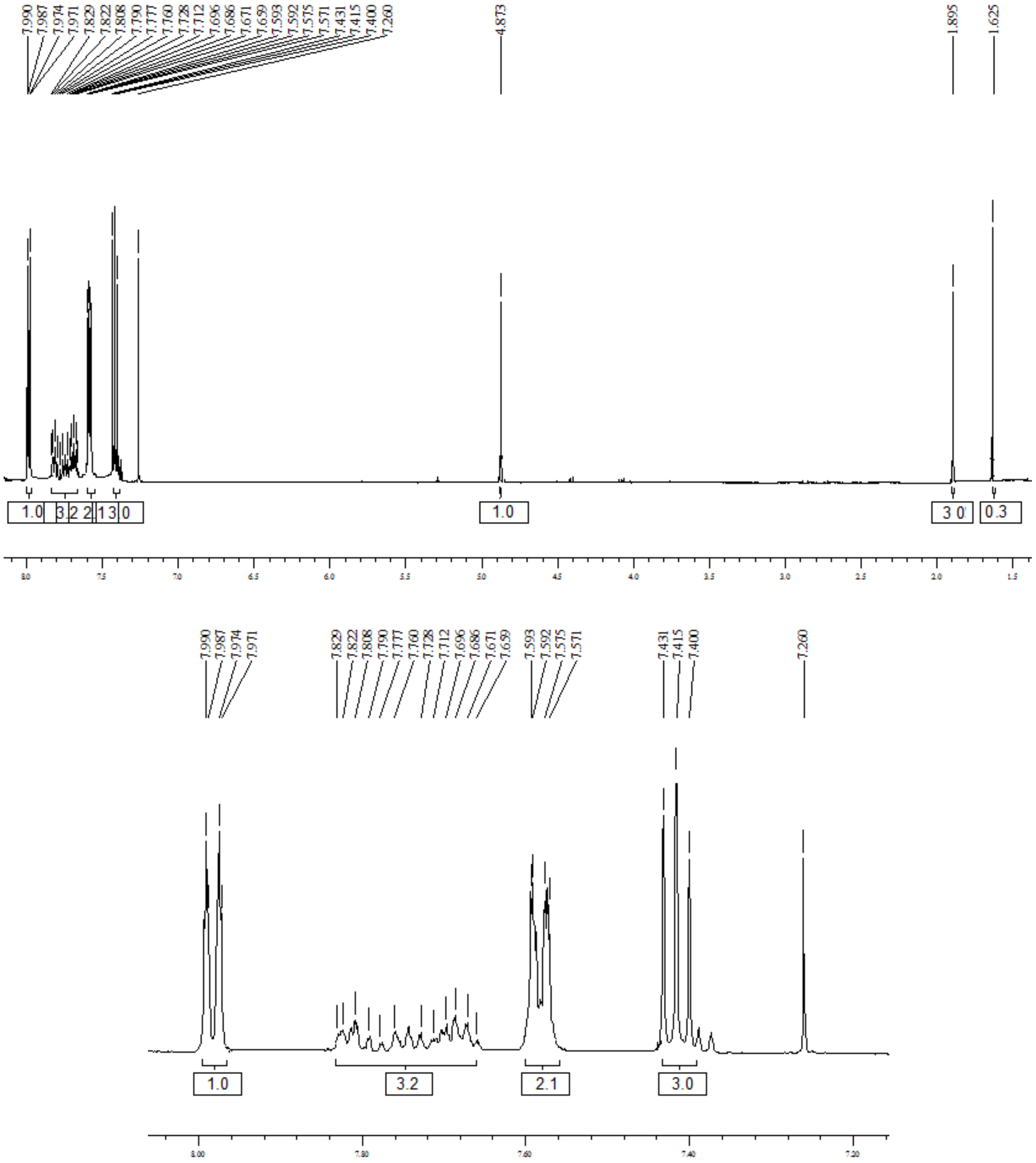

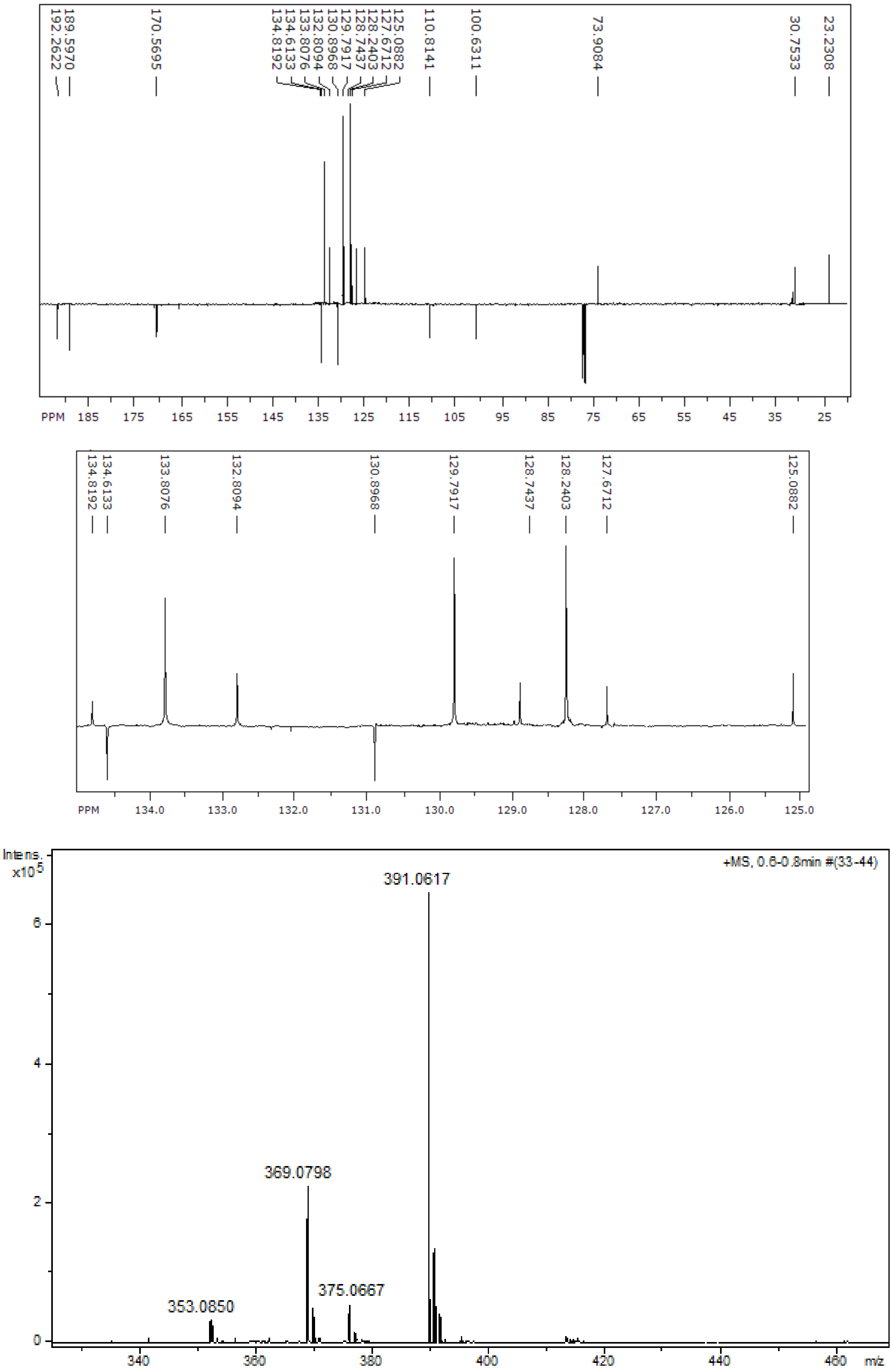

Figure 04: NMR and mass spectral data of the compound **7d**

2,2-dimethyl-3-m-tolylsulfonyl-2,3-dihydronaphtho[1,2-b]furan-4,5-dione **(7e)**

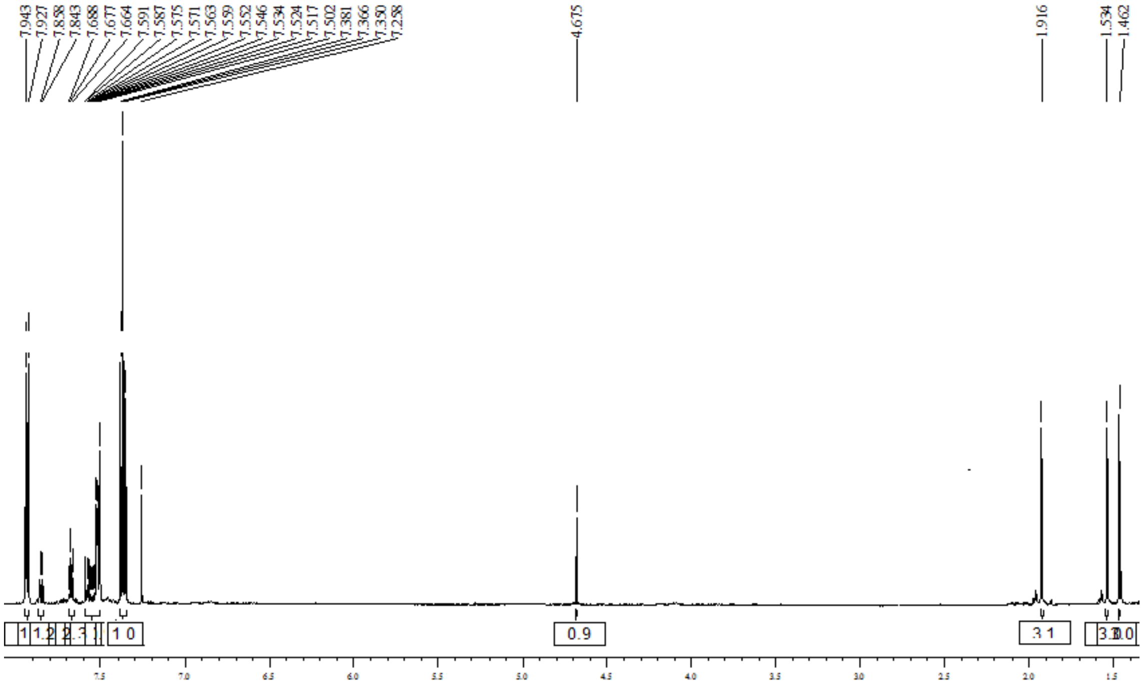

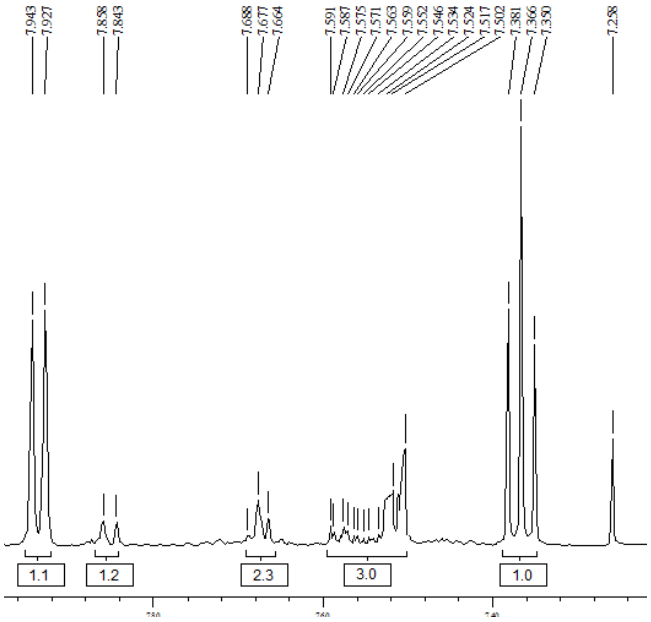

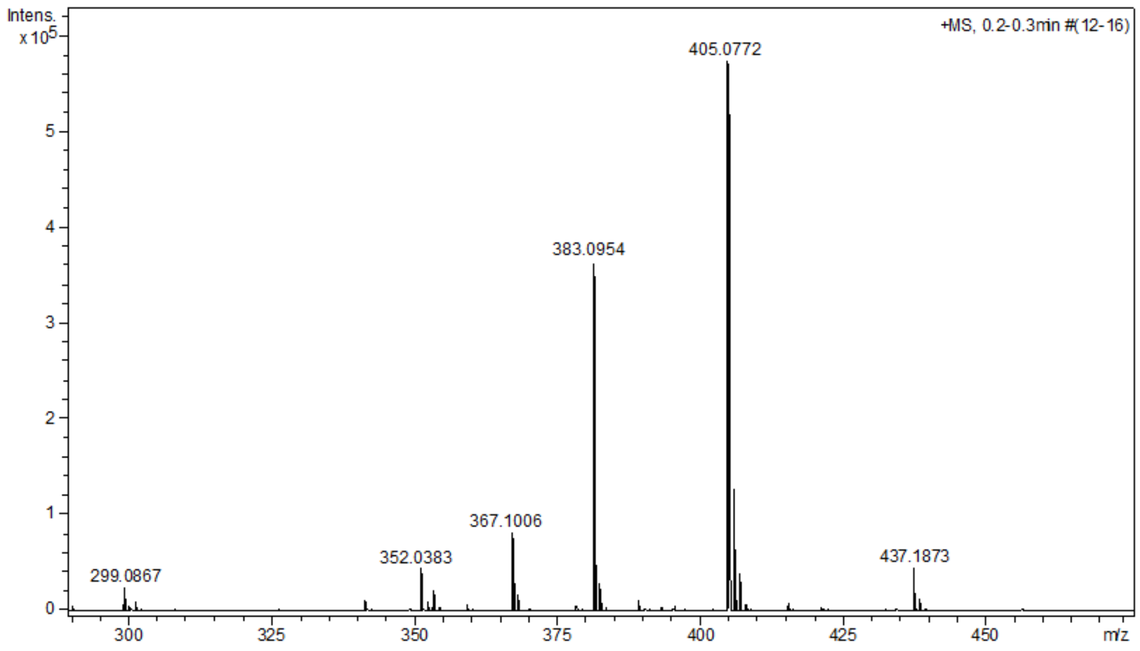

Figure 05: NMR and mass spectral data of the compound **7e**

3-pentafluorophenylsulfonyl-2,2-dimethyl-2,3-dihydronaphtho[1,2-b]furan-4,5-dione **(7f)**

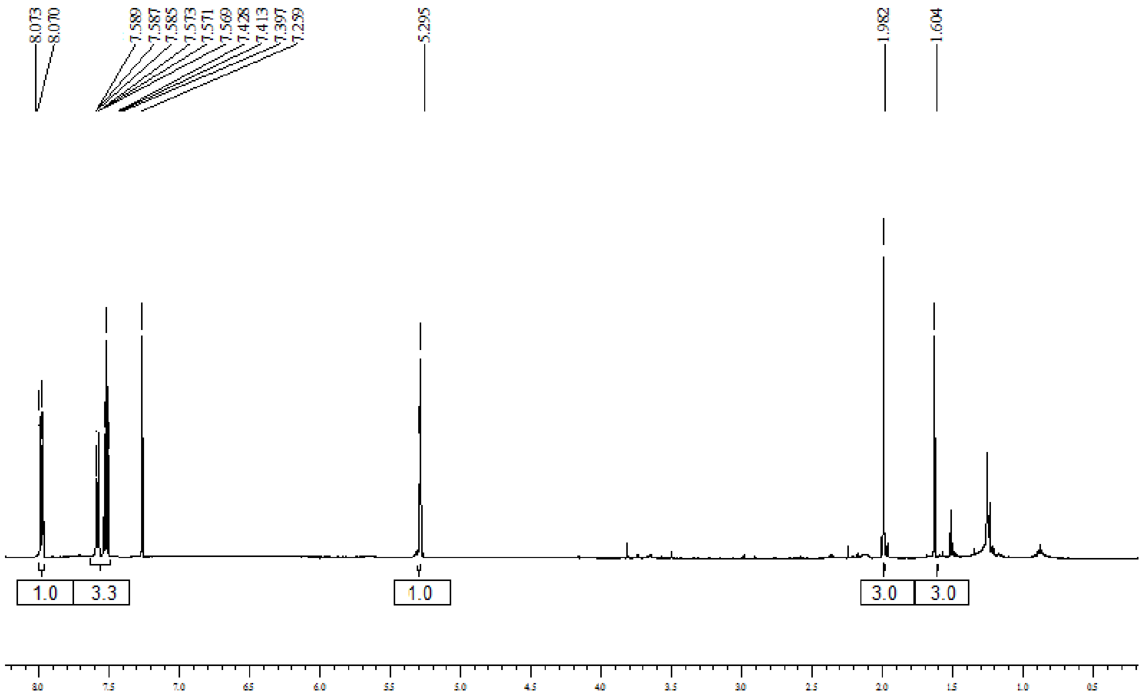

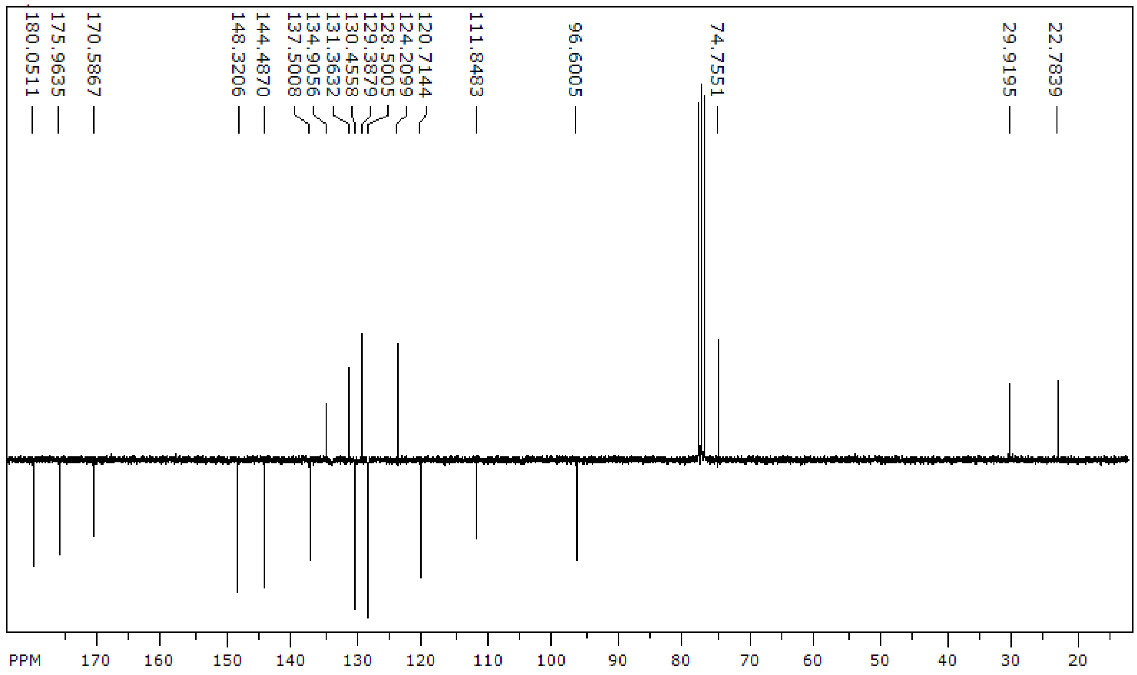

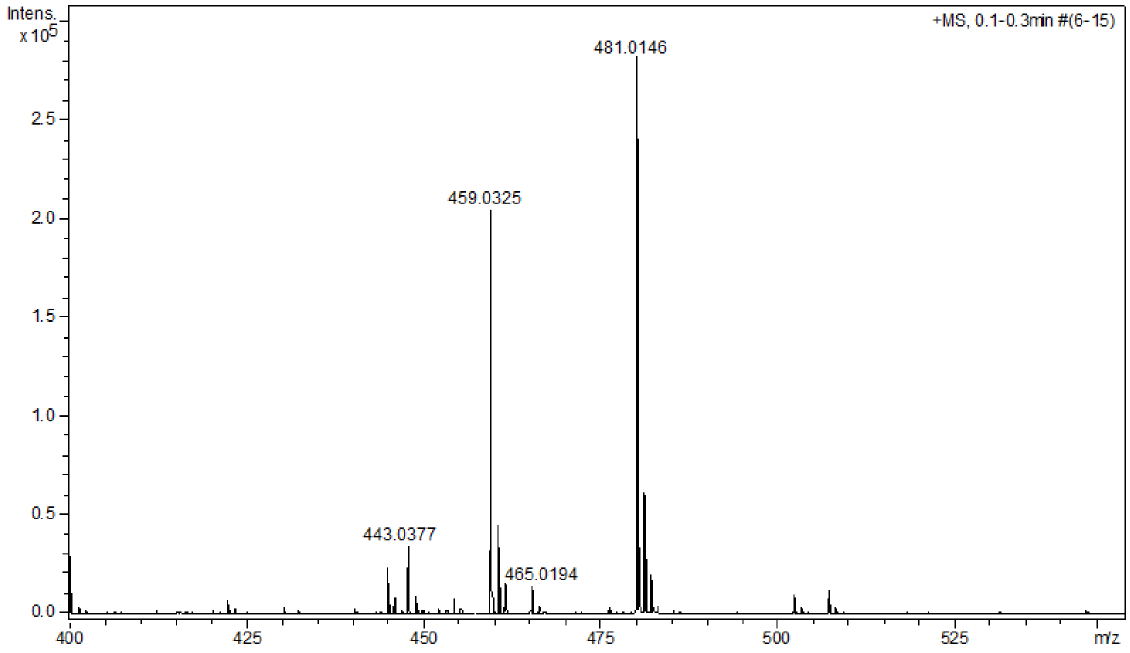

Figure 06: NMR and mass spectral data of the compound **7f**

3-cyclohexylsulfonyl-2,2-dimethyl-2,3-dihydronaphtho[1,2-b]furan-4,5-dione **(7g)**

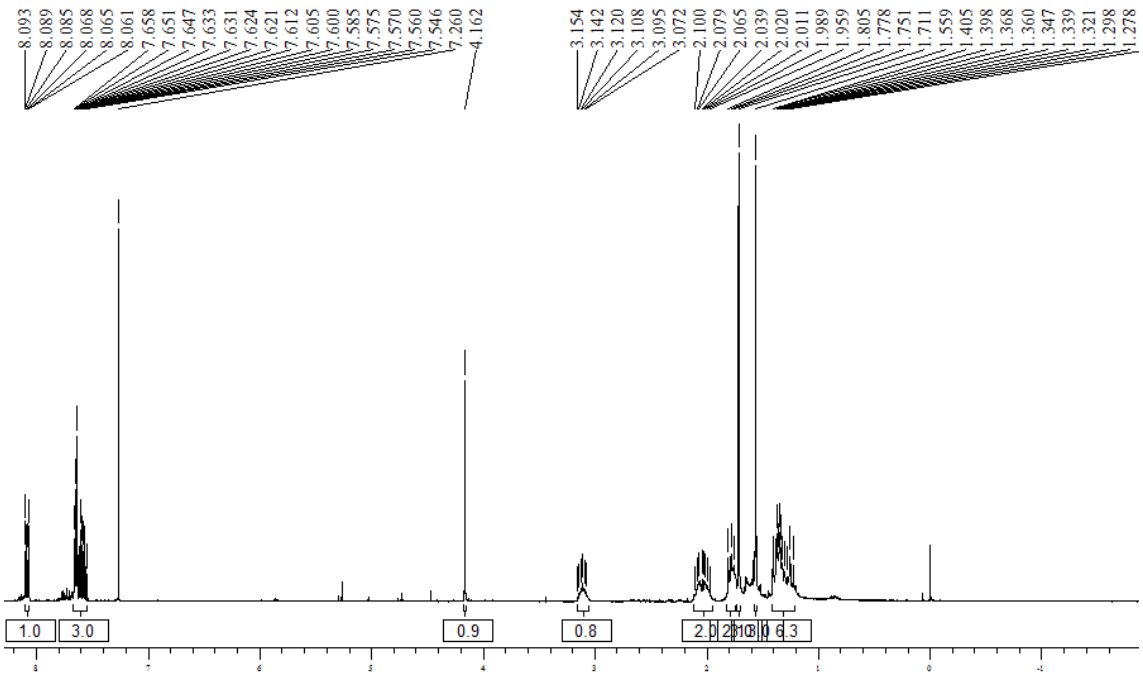

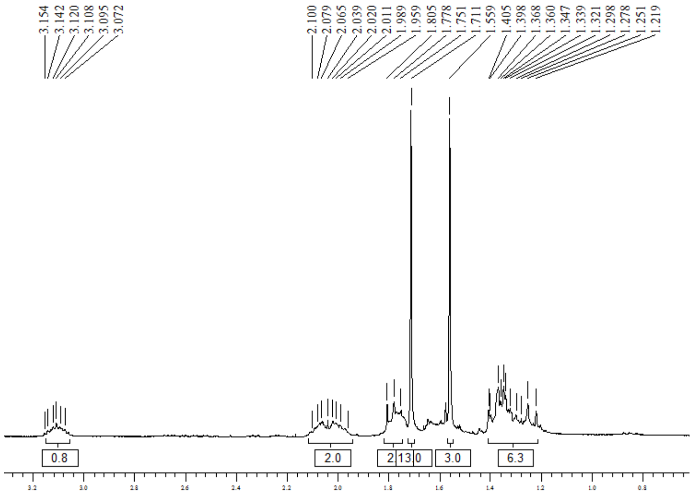

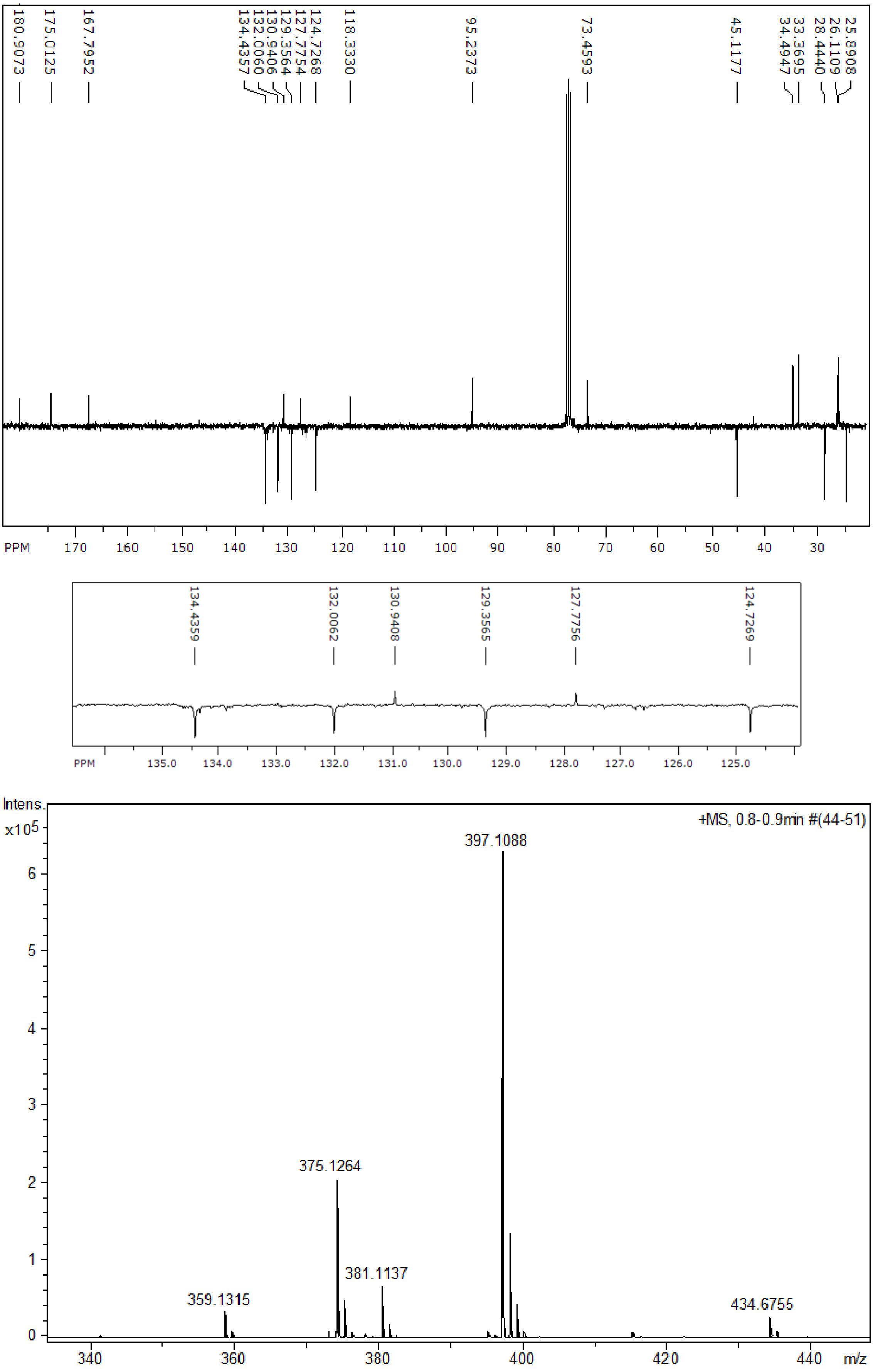

Figure 07: NMR and mass spectral data of the compound **7g**

*3-(Cyclohexylthio)-2,2-dimethyl-2,3-dihydronaphtho[1,2-b]furan-4,5-dione (**4g**)*

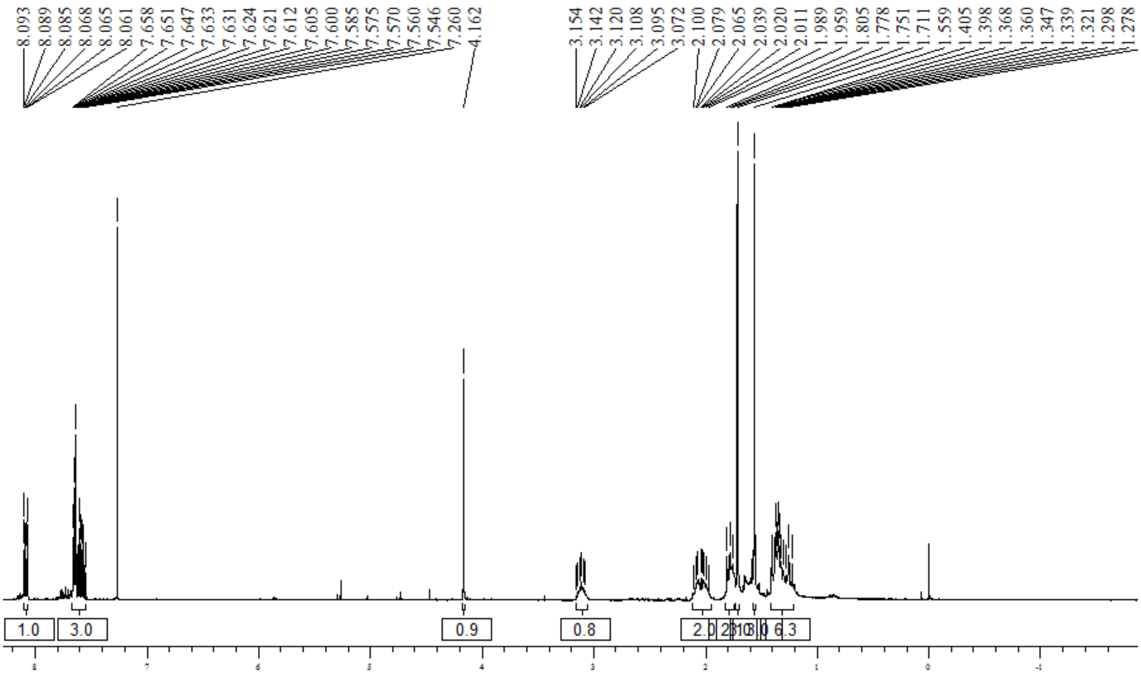

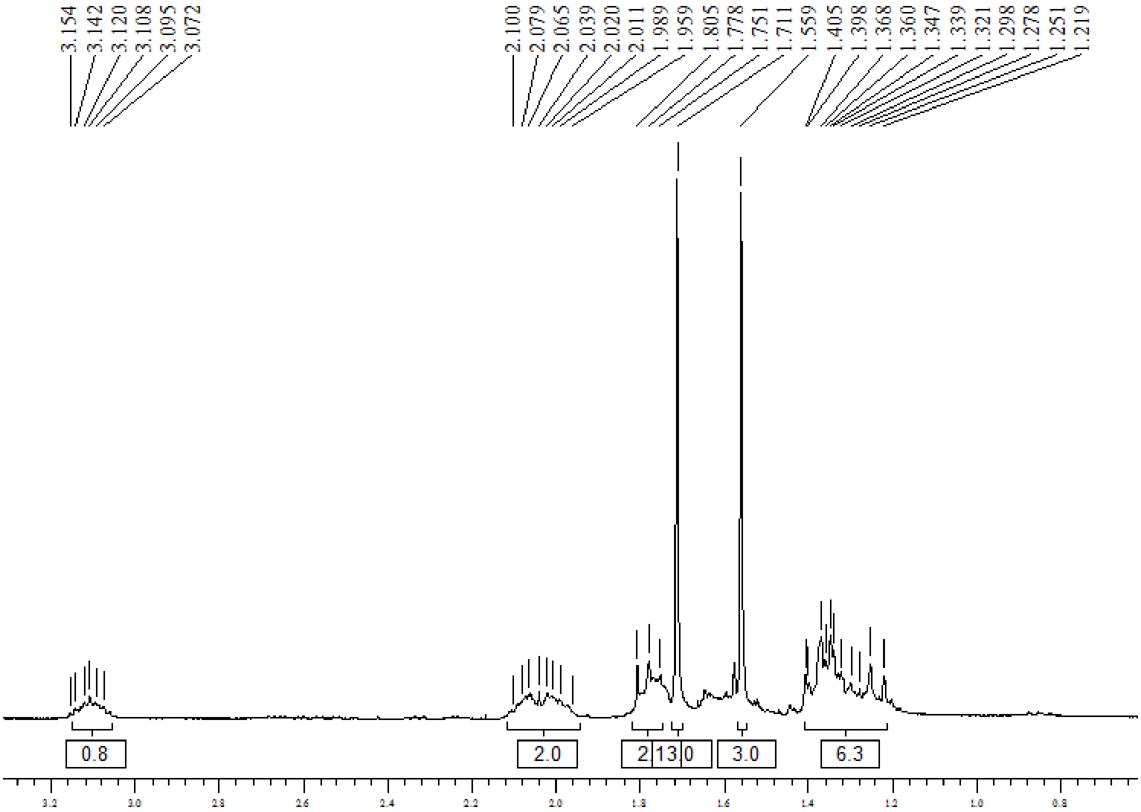

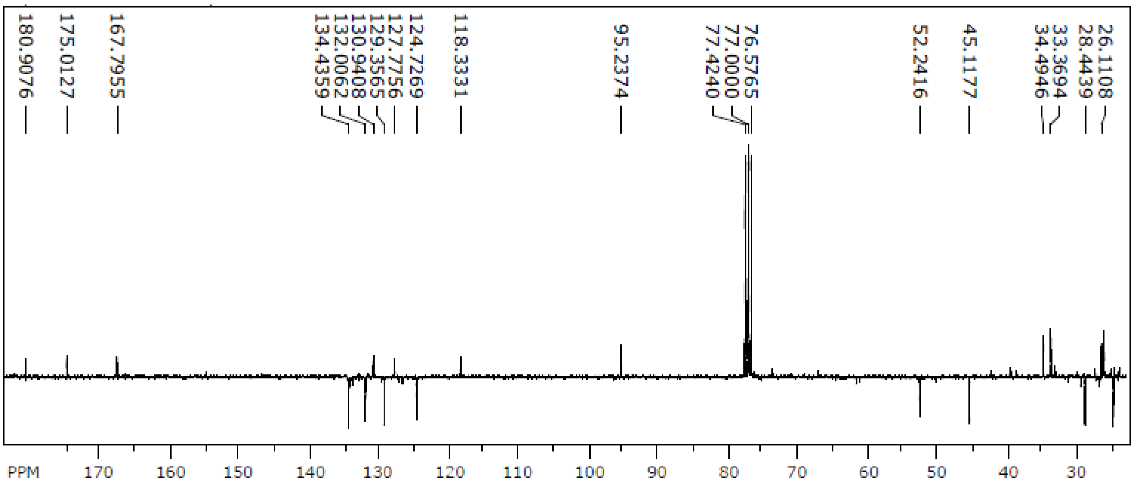
180.9076
175.0127
167.7955
134.4359
132.0062
130.9408
129.3565
127.7756
124.7269
118.3331
95.2374
77.4240
77.0000
76.5765
52.2416
45.1177
34.4946
33.3694
28.4439
26.1108
PPM 170 160 150 140 130 120 110 100 90 80 70 60 50 40 30

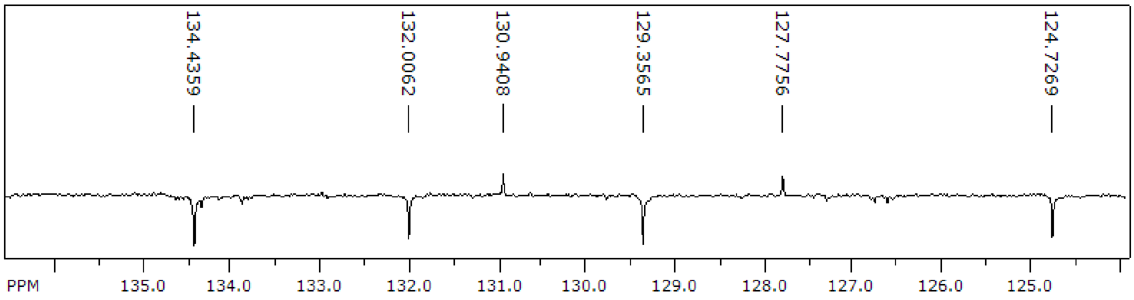
134.4359
132.0062
130.9408
129.3565
127.7756
124.7269
PPM 135.0 134.0 133.0 132.0 131.0 130.0 129.0 128.0 127.0 126.0 125.0

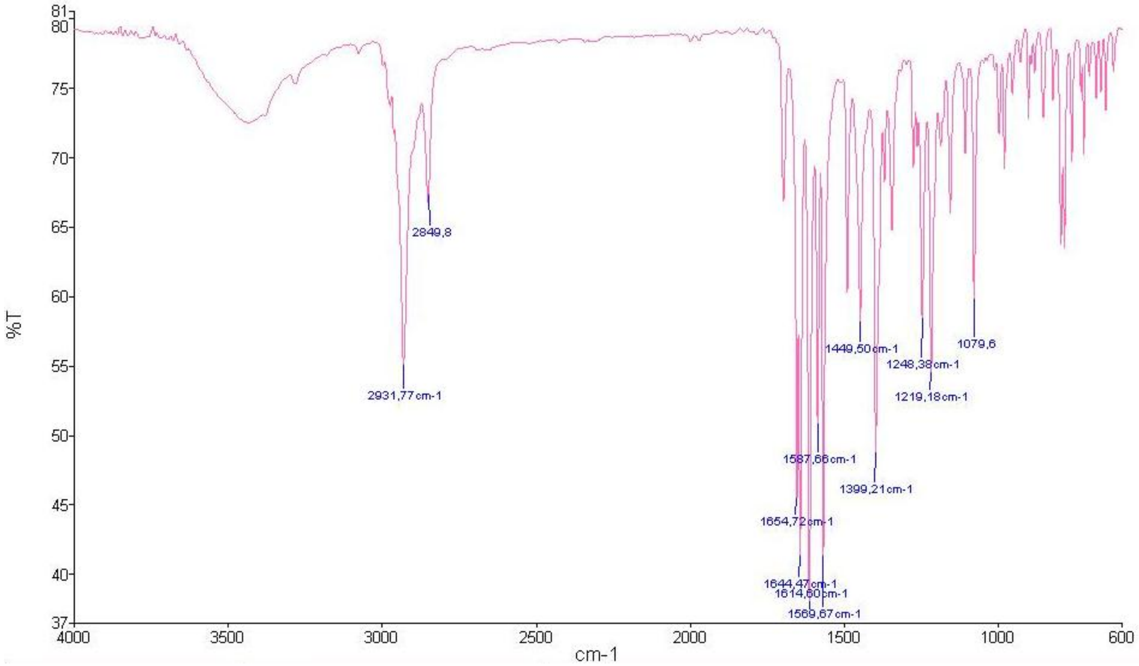
%T
cm-1
2849,8
2931,77 cm-1
1449,50 cm-1
1248,38 cm-1
1219,18 cm-1
1079,6
1587,66 cm-1
1399,21 cm-1
1654,72 cm-1
1644,47 cm-1
1614,60 cm-1
1569,67 cm-1

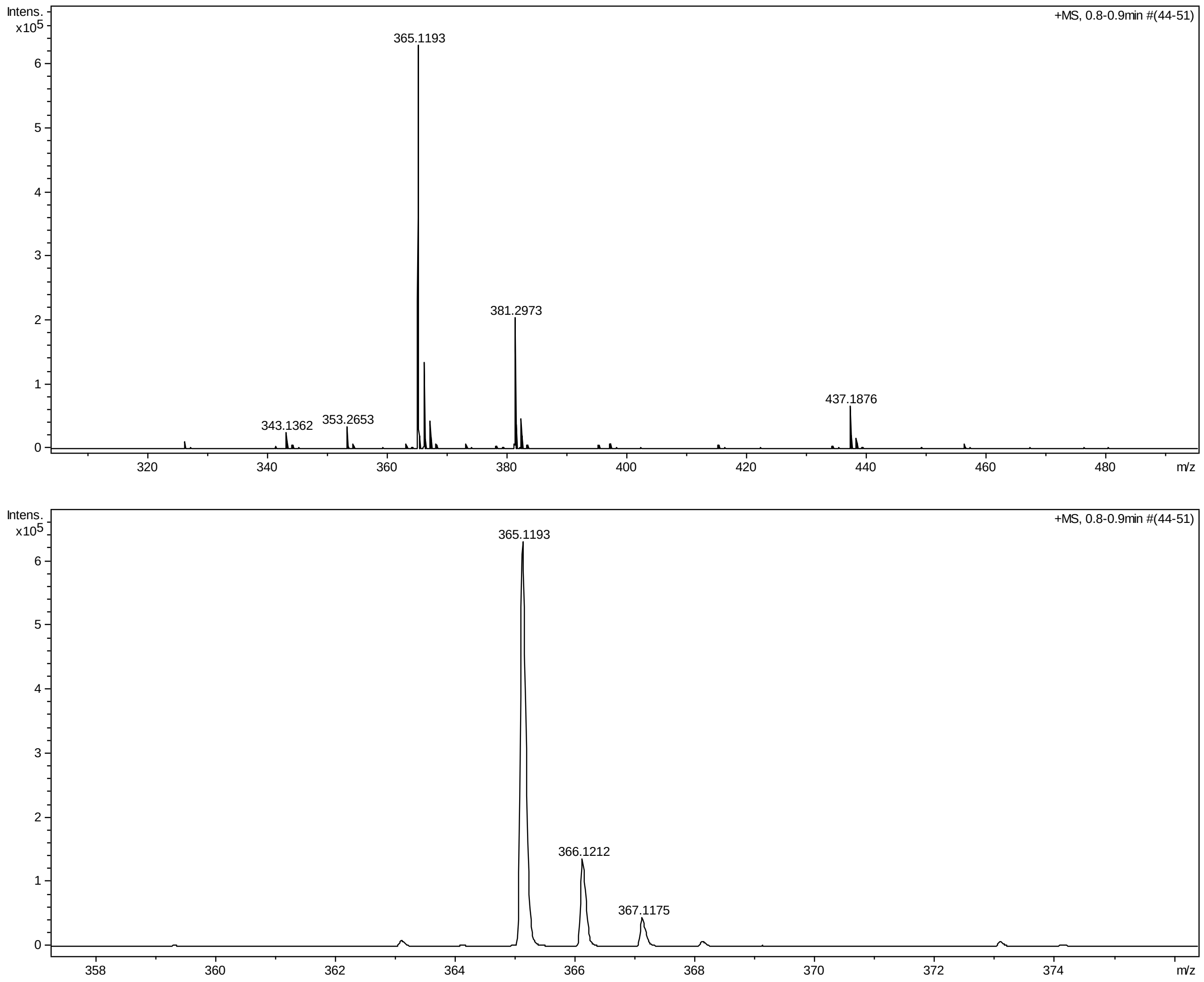

Figure 08: NMR and mass spectral data of the compound **4g**
